# Identification and Temporal Expression Analysis of Conserved and Novel MicroRNAs in the Leaves of Winter Wheat Grown in the Field

**DOI:** 10.3389/fgene.2019.00779

**Published:** 2019-09-04

**Authors:** Yong-Fang Li, Kangning Wei, Menglei Wang, Li Wang, Junxia Cui, Daijing Zhang, Junqiang Guo, Miao Zhao, Yun Zheng

**Affiliations:** ^1^College of Life Sciences, Henan Normal University, Xinxiang, China; ^2^Faculty of Information Engineering and Automation, Kunming University of Science and Technology, Kunming, China; ^3^Yunnan Key Laboratory of Primate Biomedical Research, Institute of Primate Translational Medicine, Kunming University of Science and Technology, Kunming, China

**Keywords:** winter wheat, miRNAs, expression, growth and development, target gene

## Abstract

Cold acclimation and vegetative/reproductive transition are two important evolutionary adaptive mechanisms for winter wheat surviving the freezing temperature in winter and successful seeds setting in the next year. MicroRNA (miRNA) is a class of regulatory small RNAs (sRNAs), which plays critical roles in the growth and development of plants. However, the regulation mechanism of miRNAs during cold acclimation and vegetative/reproductive transition of winter wheat is not much understood. In this study, four sRNA libraries from leaves of winter wheat grown in the field at the three-leaf stage, winter dormancy stage, spring green-up stage, and jointing stage were analyzed to identify known and novel miRNAs and to understand their potential roles in the growth and development of winter wheat. We examined miRNA expression using a high-throughput sequencing technique. A total of 373 known, 55 novel, and 27 putative novel miRNAs were identified. Ninety-one miRNAs were found to be differentially expressed at the four stages. Among them, the expression of six known and eight novel miRNAs was significantly suppressed at the winter dormancy stage, whereas the expression levels of seven known and eight novel miRNAs were induced at this stage; three known miRNAs and three novel miRNAs were significantly induced at the spring green-up stage; six known miRNAs were induced at the spring green-up stage and reached the highest expression level at the jointing stage; and 20 known miRNAs and 10 novel miRNAs were significantly induced at the jointing stage. Expression of a number of representative differentially expressed miRNAs was verified using quantitative real-time polymerase chain reaction (qRT-PCR). Potential target genes for known and novel miRNAs were predicted. Moreover, six novel target genes for four *Pooideae* species-specific miRNAs and two novel miRNAs were verified using the RNA ligase-mediated 5′-rapid amplification of cDNA ends (RLM-5’RACE) technique. These results indicate that miRNAs are key non-coding regulatory factors modulating the growth and development of wheat. Our study provides valuable information for in-depth understanding of the regulatory mechanism of miRNAs in cold acclimation and vegetative/reproductive transition of winter wheat grown in the field.

## Introduction

Flowering at favorable conditions is an important adaptive trait for reproductive success. Plants have evolved to transit from the vegetative to the reproductive growth phase at a particular time of year to ensure maximal reproductive success in a given region (Amasino and Michaels, 2010; Bouché et al., 2017). Generally, wheat cultivars are divided into two types: winter wheat and spring wheat; winter wheat requires prolonged exposure to cold temperatures to accelerate flowering (vernalization), whereas spring wheat does not require vernalization. Vernalization is regulated by diverse genetic and epigenetic networks (Xu and Chong, 2018). The roles of and interaction among several key vernalization associated genes, like *VRN1*, *VRN2*, *VRN3*, *VER2* (a carbohydrate-binding protein) and wheat *GRP2* (an RNA-binding protein), have been studied considerably well at gene expression and protein interaction levels (Danyluk et al., 2003; Yan et al., 2003; Yan et al., 2006; Oliver et al., 2013; Xiao et al., 2014). Under natural growth conditions, seeds of winter wheat are usually sown in the autumn; therefore, cold acclimation and vegetative/reproductive transition are two important evolutionary adaptive mechanisms for winter wheat surviving the freezing temperature in winter and successful seeds setting in the next year. The molecular basis of cold acclimation in plants has been extensively studied in *Arabidopsis*, wheat, and barley (Thomashow, 1999; Kim et al., 2009; Thomashow, 2010; Vashegyi et al., 2013; Li et al., 2018). However, little is known about the regulatory mechanism of small RNAs (sRNAs), especially microRNAs (miRNAs), during cold acclimation and vegetative/reproductive transition of winter wheat grown in the field.

MicroRNAs are single-stranded, ∼20- to 22-nt-long RNA molecules, which have emerged as a prominent class of regulatory RNAs. They regulate target gene expression post-transcriptionally through sequence complementary dependent cleavage or translational inhibition (Llave et al., 2002; Rogers and Chen, 2013; Yu et al., 2017). The critical role of miRNAs in developmental processes is reflected not only in pleiotropic developmental defects associated with miRNA biogenesis mutants, *dcl1*, *hyl1*, *hen1*, *se1*, and *ago1*, but also in the fact that considerable data are available for the involvement of miRNAs in tissue and organ development in several plant species (Yu et al., 2017). miR156 is highly expressed early in juvenile plants and decreases as they transition to the reproductive stage, whereas miR172 shows an inverse temporal expression pattern (Poethig, 2009; Wu et al., 2009). Most squamosa promoter binding-like (SPL) transcription factors (TFs) in *Arabidopsis* are regulated by miR156 and inﬂuence plant developmental processes, such as phase transition from the juvenile to the adult stage, root development, and leaf morphology (Wu and Poethig, 2006; Gao et al., 2017). SPL9 directly activates the expression of miR172 to facilitate ﬂowering by suppressing the APETALA2 (AP2) protein (Aukerman and Sakai, 2003; Wu et al., 2009; Zhu and Helliwell, 2011). Overexpression of miR156 in *Arabidopsis*, switchgrass, rice, maize, and Chinese cabbage was reported to prolong the juvenile phase and increase the total leaf/tiller numbers and biomass but resulted in delayed ﬂowering (Wu and Poethig, 2006; Schwarz et al., 2008; Wang et al., 2009; Fu et al., 2012). Elevated expression of miR159 in *Arabidopsis* resulted in a delay in flowering under a short-day photoperiod through its effects on gibberellin- and abscisic acid-regulated MYB (GAMYB) activity (Achard et al., 2004). miR396 regulates the expression of growth regulating factor (GRF) genes involved in the control of cell proliferation during leaf and root development (Rodriguez et al., 2010; Bazin et al., 2013). Increasing evidence indicates that miRNAs are involved in the regulation of seed/grain development in crop plants. In rice, OsSPL16/GW8, targeted by miR156, controls the size, shape, and quality of grains by promoting cell division and grain filling (Wang et al., 2012a). Overexpression of miR397 could improve the yield in rice plants by increasing the grain size and promoting panicle branching (Zhang et al., 2013).

In wheat, about a dozen studies have been performed on miRNA identification using sRNA sequencing (sRNA-Seq) (Yao et al., 2007; Wei et al., 2009; Li et al., 2013; Meng et al., 2013; De Paola et al., 2016; Fileccia et al., 2017; Wang et al., 2018). miRNAs responding to diverse stresses in wheat have also been reported (Xin et al., 2010; Zhou et al., 2015; Akdogan et al., 2016; Chen et al., 2017; Zuluaga et al., 2017; Han et al., 2018). miRNAs related to the development and filling of wheat seeds have been studied (Meng et al., 2013; Han et al., 2014; Li et al., 2015; Wang et al., 2018). For example, Meng et al. (2013) identified 104 miRNAs associated with grain filling in wheat. Han et al. (2014) found that 4 known miRNA families and 22 novel miRNAs were preferentially expressed in developing seeds. Little is known about miRNAs and their regulatory functions in the cold acclimation and vegetative/reproductive transition of winter wheat grown in the field under natural weather conditions. Therefore, this study aimed to systematically identify the conserved and novel miRNAs in wheat leaves at different growth stages under natural growth conditions. To achieve this, we sequenced four sRNA libraries prepared from wheat leaves at the three-leaf stage, winter dormancy stage, spring green-up stage, and jointing stage. This study provides useful information for uncovering the regulatory networks of miRNAs in wheat leaves at different growth and developmental stages.

## Materials and Methods

### Plant Materials

Wheat (*Triticum aestivum* L.) variety Shimai 22 was used in this study. Shimai 22 is a high-yield semi-winter wheat variety bred by Shijiazhuang Academy of Agriculture and Forestry (Shijiazhuang, Hebei, China) and is widely grown in the northern region of the Huang-Hai plain (Fu et al., 2016). Wheat seeds were planted on October 1, 2015, at the Wheat Experimental Station of Henan Normal University (35°19′ N, 113°54′ E). The top two fully expanded leaves were collected at the three-leaf stage before the cold winter (Leaf_1, October 24, 2015), winter dormancy stage (Leaf_2, January 7, 2016), spring green-up stage or double ridge stage (Leaf_3, March 4, 2016), and jointing stage (Leaf_4, April 10, 2016). Three independent samples were collected and used as biological replicates. All the samples were collected at the same time (between 1:30 and 3:00 PM) to minimize the circadian rhythm effect, snap-frozen in liquid nitrogen, and stored at −80°C until RNA isolation.

### Construction and Sequencing of Small RNA Library

Total RNA was extracted using TRIzol reagent (Invitrogen, USA), according to the manufacturer’s instructions. The integrity of the RNA samples was checked using 1% agarose gel electrophoresis and further checked by Agilent Bioanalyzer 2100. Equal amounts of total RNA samples from three biological replicates were mixed and sent to BGI (Shenzhen, China) for construction of sRNA libraries. Small RNAs, ranging in length from 18 to 30 nt, were enriched and purified by 15% denatured polyacrylamide gel electrophoresis (PAGE). Thereafter, 3′- and 5′-RNA adaptors were ligated to sRNAs. This was followed by reverse transcription and final polymerase chain reaction (PCR) enrichment. The sRNA libraries were PAGE-purified (Wang et al., 2015). High-throughput sequencing of the four sRNA libraries was performed on a BGISEQ500RS platform.

### Bioinformatics Analysis of sRNA Sequence Data

Raw reads generated by deep sequencing were analyzed, as described previously (Jagadeeswaran et al., 2012; Li et al., 2013; Zheng et al., 2016). Briefly, raw reads were first filtered by removing low-quality reads and trimming the adaptor sequences, and clean reads (18–30 nt) were obtained for each sRNA library. Identical reads were pooled to generate non-redundant sequences (unique reads). Small RNAs originating from rRNAs, tRNAs, small nuclear RNAs (snRNAs), small nucleolar RNAs (snoRNAs), and repeat RNAs were further removed by alignment against database Rfam (r12) (Nawrocki et al., 2015), NONCODE (v3.0) (Bu et al., 2012), GtRNAdb (Chan and Lowe, 2008), Plant Repeat Databases (Bao et al., 2015), and Repbase (r20) (Bao et al., 2015) using SOAP (Li et al., 2009). The remaining sRNAs were first used to identify the known miRNAs. Sequences of mature miRNAs from all plant species were downloaded from miRBase Release 21.0 (http://www.mirbase.org) and combined to obtain all known plant miRNA sequences. The known miRNAs in wheat leaves were identified by BLAST search against all known plant miRNA sequences. Small RNAs with fewer than two mismatches with known plant miRNAs were considered as the known wheat miRNAs. The remaining sRNAs were used to identify novel wheat miRNAs using MIREAP software (http://sourceforge.net/projects/mireap/). They were first mapped to the genome of Chinese Spring wheat (ftp://ftp.ensemblgenomes.org/pub/plants/release-31/fasta/triticum_aestivum/), and only perfectly matched sRNAs were used for further analysis. The flanking regions (150 nt upstream and 150 nt downstream) of sRNAs were isolated and used to predict fold-back structure with the RNAfold software (http://rna.tbi.univie.ac.at/cgi-bin/RNAWebSuite/RNAfold.cgi). The fold-back structures of novel miRNAs were further checked manually using the Mfold Web Server (Zuker, 2003). The key criteria for novel miRNA annotation were based on the ones described in a recent article (Axtell and Meyers, 2018). Only those miRNAs with an abundance of more than 50 reads in at least one leaf sample together with detected miRNA* were considered as novel miRNAs. Those for which miRNA* were not detected but that could form a typical miRNA precursor fold-back structure were considered as wheat putative miRNAs.

### Identification and Validation of Differentially Expressed miRNAs

To quantify miRNA expression, tags per million reads (TPM) was used to normalize the miRNA expression levels. We first compared the expression patterns of the identified miRNAs at the four different growth and development stages using GENESIS (Sturn et al., 2002). Thereafter, DEGseq was used to screen the differentially expressed miRNAs (Wang et al., 2010). MicroRNAs with fold change ≥ 2 between samples and *p*-value less than 0.01 were defined as differentially expressed miRNAs (DEG miRNAs). Quantitative real-time PCR (qRT-PCR) was performed to validate the expression patterns of miRNAs. Briefly, total RNA, including sRNAs, was first polyadenylated and then reverse-transcribed with poly (T) oligonucleotide into cDNA using a Mir-X miRNA qRT-PCR SYBR Kit (Takara Co., Tokyo, Japan), following the manufacturer’s instructions. Quantitative PCR was carried out using a SYBR Green (Takara) qPCR assay and LightCycler^®^ 96 Real-Time PCR System (Roche). miRNA-specific forward primer was designed based on the miRNA sequence. The sequences of these primers are listed in **Supplemental Table S1**. The universal reverse primer was provided in the Mir-X miRNA qRT-PCR SYBR Kit. All reactions were run in triplicates; U6 was used as the reference gene. Dissociation curves were checked to determine the presence of nonspecific products, and only primers resulting in a single peak and an amplicon of the correct size, as verified on an agarose gel, were used to evaluate the expression of miRNAs. The expression level of miRNAs at the three-leaf stage (Leaf_1) was set as 1.0, and the relative expression levels of miRNAs at other stages were calculated using the 2^-ΔΔCT^ method (Livak and Schmittgen, 2001).

### Prediction and Validation of the Targets of Wheat miRNAs

The potential targets of wheat miRNAs were predicted using the PsRobot pipeline (http://omicslab.genetics.ac.cn/psRobot/). Only those targets with alignment scores between the query miRNA and targets less than 2.5 were considered. RNA ligase-mediated 5′-rapid amplification of cDNA ends (RLM-5′RACE) was carried out to validate the predicted target genes using a GeneRacer kit (Invitrogen, USA). Total RNA was treated with DNase I; RNA oligo adaptor was directly ligated to total RNA, and GeneRacer oligo dT primer and Superscript II reverse transcriptase (Invitrogen, USA) were used to synthesize the first-strand cDNAs. This was followed by nested PCR and electrophoresis of PCR products on a 2% agarose gel. The bands with expected sizes were purified using a QIAquick^Ⓡ^ Gel Extraction Kit (QIAGEN) and ligated to pMD19-T (Simple) vector (Takara). The ligation was transformed into *Escherichia coli* DH5α competent cells, and positive colonies identified using colony PCR were used for isolation of plasmids, which were subjected to Sanger sequencing (Li et al., 2010).

## Results

### Overview of sRNA Library Sequencing Data

**Figure 1:**
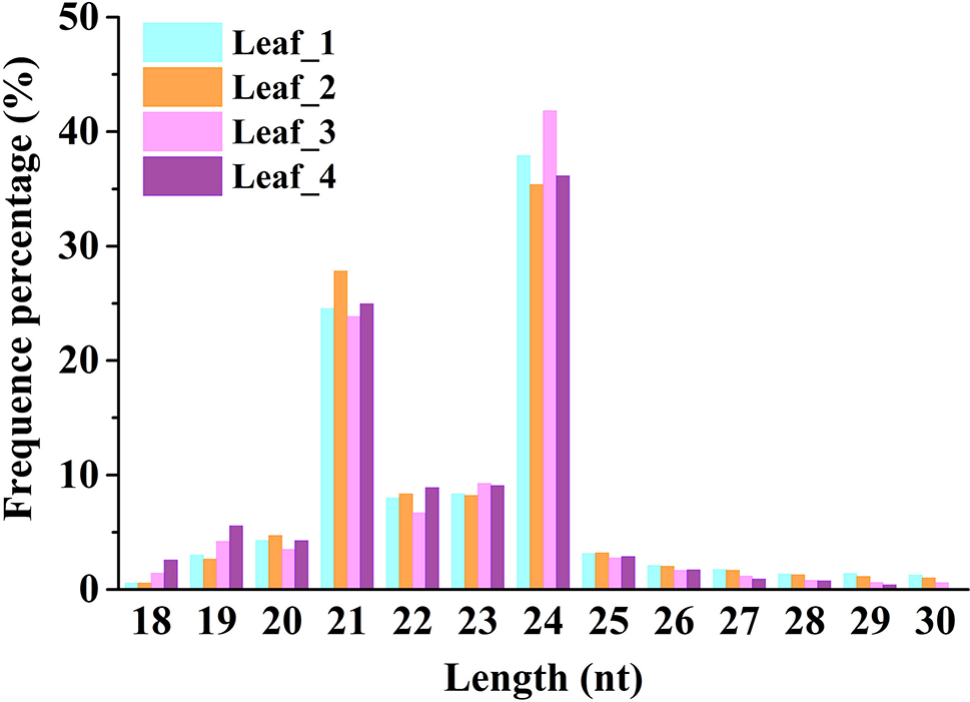
Size distribution of small RNAs in wheat leaves at four different growth and development stages. Leaf_1, Leaf_2, Leaf_3, and Leaf_4 represent wheat leaves collected at the three-leaf stage, winter dormancy stage, spring green-up stage, and jointing stage, respectively.

To study the involvement of regulatory miRNAs in wheat leaves during cold acclimation and vegetative/reproductive transition, four sRNA libraries were generated and sequenced. The four libraries represent the four growth stages of winter wheat grown in the field under natural weather conditions, viz. three-leaf stage, winter dormancy stage, spring green-up stage, and jointing stage. All sequencing data were first processed by filtering low-quality reads and trimming adaptor sequences, and clean reads were generated for each sRNA library. Identical reads were subsequently pooled to create unique reads. A total of 90,372,481 clean reads representing 36,634,063 unique reads, ranging in length from 18 to 30 nt, were obtained for further analysis. The length distribution of sRNA libraries is summarized in **Figure 1**. All four libraries shared a similar distribution pattern. The most abundant sRNA reads were 24 nt long (about 30%), followed by reads of 21 nt length (15%), which is the typical length of canonical miRNAs (**Figure 1**). The patterns were consistent with the typical read distribution reported in other studies. The percentage of reads mapping to wheat genome was >87.82%, and reads mapping to tRNAs, rRNAs, snoRNAs, and snRNAs accounted for ∼8.35%–14.42% of the total reads, indicating a high quality of all the sRNA libraries prepared (**Table 1**).

**Table 1:** Read statistics of four wheat leaf small RNA libraries.

Type	Raw reads	Clean reads	Mapped reads	rRNA	snRNA	snoRNA	tRNA	Unique reads	Unique reads mapped
Leaf_1	23,609,453	22526694 (100%)	20300798 (90.12%)	2643342 (11.74%)	8960 (0.04%)	3684 (0.02%)	485426 (2.15%)	9355147 (100%)	7915731 (84.61%)
Leaf_2	23,497,910	22543808 (100%)	20548305 (91.15%)	2591106 (11.49%)	6251 (0.03%)	1931 (0.01%)	425319 (1.89%)	8201915 (100%)	6935745 (84.56%)
Leaf_3	21,410,747	20444752 (100%)	18127870 (88.67%)	1290962 (6.31%)	3670 (0.02%)	936 (0.005%)	408480 (2%)	8877395 (100%)	7377298 (83.10%)
Leaf_4	25,877,348	24857227 (100%)	21830151 (87.82%)	1570443 (6.32%)	16601 (0.07%)	3007 (0.01%)	768556 (3.09%)	10199606 (100%)	8090818 (79.32%)
Total	94,395,458	90,372,481 (100%)	80,807,124	8,095,853	35,482	9,558	2,087,781	36,634,063	30,319,592

### Identification of Known miRNAs in Wheat Leaves

For identifying the known miRNAs in wheat, we first downloaded the mature miRNA sequences from all plant species present in miRBase and combined them to obtain all known plant miRNA sequences. The unique reads from the four wheat sRNA libraries were aligned with all known plant miRNA sequences using BLASTN search. The unique reads that matched the known plant miRNA sequences with ≤2 mismatches were annotated as known wheat miRNAs. A total of 373 known miRNAs belonging to 289 miRNA families were identified from the four libraries. The 24 most conserved plant miRNAs (miR156/157, miR159, miR160, miR161, miR162, miR164, miR165/166, miR167, miR168, miR169, miR170/171, miR172, miR319, miR390, miR391, miR393, miR394, miR395, miR396, miR397, miR398, miR399, miR408, and miR444) were identified among these known miRNAs. Wheat has 125 mature miRNAs belonging to 99 miRNA families in miRBase (Release 22.1), of which 77 miRNA families (77.8%) were found in this study, indicating the deep sequencing coverage of the four sRNA libraries.

Among the conserved miRNAs, miR168 was found to be the most abundant miRNA at all four stages (more than 109,365 TPM) and was followed in abundance by miR159, miR167, miR170/171, and miR396; miR156/157, miR164, miR165/166, miR319, miR393, and monocot-specific miR444 were also found to have high expression levels, whereas miR161, miR162, miR390, miR391, miR394, and miR408 showed relatively low expression levels. Among the less conserved known miRNAs, most exhibited low expression levels, whereas miR5048, miR5062, miR5200, miR7757, miR9962, miR9672b, miR9674, and miR9773 were expressed at high levels in wheat leaves at all four growth and development stages (**Supplemental Table S2**). These data indicate that expression of miRNAs in leaf tissues at different growth stages varies greatly among the different miRNA families.

### Identification of Novel miRNAs From Wheat Leaves

High-throughput sequencing has the potential for detecting novel miRNAs with low expression in the sRNA transcriptome. The remaining sRNA sequences that did not have any homologs with known plant miRNAs were used to predict potential novel miRNAs using MIREAP software. Only those sRNAs with more than 50 reads in at least one library were used for further analysis. We manually checked the hairpin structure of the predicted novel miRNAs by Mfold software (Zuker, 2003), and only those with the typical stem-loop structure were considered (**Supplemental File S1**–**S2**). A total of 55 novel miRNAs, for which corresponding miRNAs* were detected, were identified. The length of these novel miRNAs and miRNAs* varied from 20 to 23 nt (**Table 2**). The locations of their precursors and their sequences are listed in **Supplemental Table S3**. Among them, novel_miR127, novel_miR128, novel_miR390, novel_miR1544, novel_miR1560, and novel_miR1686 were the most abundantly expressed miRNAs, and their expression levels were higher than those of some conserved miRNAs (**Supplemental Table S4**). We also assigned 27 putative novel miRNAs, and although no corresponding miRNA* was detected for them, their precursors could form the characteristic hairpin structure (**Supplemental File 2**); novel_miR1202, novel_miR527, novel_miR1327, novel_miR1620, novel_miR1356, and novel_miR1615 were relatively abundant putative miRNAs identified in wheat leaves (**Supplemental Table S5**). The information of the putative miRNA precursors and their chromosome location are shown in **Supplemental Table S6**.

**Table 2:** Expression of novel miRNAs.

miR_name	miRNA location	miRNA expression (TPM)				miRNA sequence	miRNA length (nt)	miRNA* sequence	miRNA* length (nt)
		Leaf_1	Leaf_2	Leaf_3	Leaf_4				
novel_miR30	3p	15.3	22.9	14.2	9.2	CAUUUUCCUAUAGACUUGGUC	21	CCAAGUCUAGAGAAUAAUGUA	21
novel_miR127	5p	49.5	53.8	50.8	34.6	UUUGAAGACUAGUUUAUUACA	21	UAAUAAACAAGUCUUUAGAUG	21
novel_miR128	5p	115.0	133.8	94.4	92.1	UCUGAAGACUAGUUUAUUACA	21	UAAUAGACAAGUCUUUUGAUG	21
novel_miR193	3p	24.6	17.6	8.6	9.9	UAAGCACCUCCCCUAAGCACCU	22	AUGCUUAGGGAGGUGUUUAGA	21
novel_miR259	5p	6.5	18.8	19.1	16.2	GAGAUGACACCGACGCCGAUC	21	UCGGAGUUGGUGUCACCUCGC	21
novel_miR364	3p	1.9	2.8	4.7	2.7	UAAUGUUGCAACGUCCUGAAC	21	UCAGGACGUUGCAACAUUAAC	21
novel_miR381	5p	2.7	3.8	4.0	2.0	AUUACUUGUCGCGAAAAUGGA	21	CAUUUCCGAGACAAGUAAUUU	21
novel_miR390	3p	101.2	143.1	246.9	220.2	UCCGCGAUCAUCACGACCAAA	21	UUUGGUCGUGAUUAUCGCGGU	21
novel_miR553	3p	3.2	5.1	4.1	1.0	AAAAAAGAUGGAGUGGCAAUUC	22	AUUGCCACUUAAUACUUUUUUGC	23
novel_miR639	5p	2.7	1.5	0.6	1.4	AGUUGAAGAUGAGAUAUUGAA	21	CAAUAUCUCAUUUUCAACUAU	21
novel_miR641	3p	3.2	3.5	5.2	2.4	UUAUAUUAUGUGACAGAAGGA	21	CUUCCGUCUCAUAAUAUAAAA	21
novel_miR652	5p	1.8	1.5	2.7	2.3	GAAUUACUUGUCGCGAAAAUG	21	UUUUCGCGACAAGUAAUUCCG	21
novel_miR669	3p	4.7	6.4	12.9	3.3	AGUGGCCCAACUGCACCCUGC	21	GCAGGGUGCAGUUGGGUCACU	21
novel_miR678	5p	4.7	11.4	2.9	7.2	GAAGGGGAGCCUUGGCGCAGUGG	23	ACAUGCACCGGGCUGCCCUUUCUA	24
**novel_miR692**	3p	0.6	0.3	0	6.5	UCAGGUCGCCCCCGCUGGAGC	21	UCCUGCGGGGUCGAACUGGGA	21
novel_miR810	3p	25.2	39.5	21.3	59.3	AUGCCGUGUUGUUCUGAAAGAA	22	CUUCAGAAGAGCAUGUCAUUU	21
novel_miR866	5p	14.0	19.0	11.1	0.4	UAGUCUCGUCCUCUUGCUAAG	21	UGGCGAGGAUACGAGACUAUA	21
novel_miR1009	5p	3.9	7.2	3.6	5.8	UUCCGAAUUACUUGUCGCAGA	21	UGCGACGAGUAAGUUGGAACG	21
novel_miR1154	5p	13.3	7.4	18.7	62.2	AAAAAGAAUGACCUCAUUGUC	21	CAAUGAGAUCAUUCUUUUUAU	21
novel_miR1255	3p	11.2	13.8	10.2	0	AAGAAGAGGGUCAAGAAAGCUG	22	GCUUUCUUGAACUUCUCUUGC	21
novel_miR1304	3p	15.6	16.8	14.9	6.9	AUAUCAAGCACUCAUCGACAG	21	GCCGGUGAGUGCUUUAUAUCU	21
novel_miR1322	3p	41.0	44.0	37.8	0	UUUUUGGAUGUGCUCCUCUAG	21	AGAGAAGCACAUACAAAAAAA	21
novel_miR1372	5p	10.0	19.5	9.9	7.7	UUUCCGGUUCCACCUCGGCCGC	22	AGCCGAGUGGCACCGGUAACU	21
novel_miR1401	5p	1.8	4.1	1.8	1.2	CCCUCCGUCCCAUAAUAUAAG	21	UAUAUUAUGGAACGGGCGAA	20
novel_miR1419	3p	33.0	33.2	13.9	4.9	AAAUGCGGUUGUUGUUCCCUA	21	GGAACAACAACCGCAUUUUC	20
novel_miR1544	3p	535.9	915.8	267.9	455.0	AGUCUCUGCCAAUUCUUCGUG	21	CGAAGGAUUUGCAGAUACUCC	21
novel_miR1560	5p	757.2	777.6	0	0	UAAACCUUCACAAAUUCCCUUG	22	AGGAAUUUGUGAGGUUUACA	20
novel_miR1613	5p	1.6	3.0	6.2	1.5	UGGGCGUACGGAACACGGGUG	21	CCCGUGUUCACGUACGCCCAUC	22
novel_miR1617	5p	5.6	4.9	0	5.5	UGAAUCUUGGGAAAAAGCUG	20	GAUUUUUCACCAAUAUUCAAG	21
novel_miR1686	5p	88.9	159.6	102.2	89.1	UCAUCUGAAGACUAGUUUAUU	21	UCAUCUGAAGACUAGUUUAUU	21
novel_miR1737	3p	8.7	13.4	6.9	19.1	AUUGAACUAAGGAGGGGUGGA	21	CAACCCUCUUUAGUUCAAUCA	21
novel_miR1744	3p	4.0	5.8	3.8	0.0	AAUGACUUAUACACCGGGACG	21	UCCGGGUGUAUAAGUCAUUCG	21
novel_miR1875	3p	17.9	17.0	31.3	38.3	UAAUCUCACCUCAACAGCCGC	21	AGCCGUUGAGAUGAGAUUACC	21
novel_miR2079	5p	49.3	28.7	8.4	0	UGGUCUGUGUUUGUUUCAAAC	21	UUGAGACGAAAACAGACCAAC	21
novel_miR2133	3p	1.3	2.5	1.2	1.1	AUCCGUAUCUAGACAAAUCUAAG	23	UGGAUUUGUCUAGAUAUAGAUGU	23
novel_miR2185	3p	17.0	0.0	13.7	10.7	UCAUUUGGCAUUGCUUUCCCU	21	AGAAAGCAAUGUCAGAGGAGU	21
novel_miR2191	5p	6.1	5.6	9.2	6.5	UGAUCCCACGUCUAAGGCCUG	21	GGACUUAGAACAUGGGGUCGAC	22
novel_miR2567	5p	0	12.1	11.6	0	UCAUCAAUUUCUGUCUAACUA	21	GUUAGACAGAAAUUGAUGAAG	21
novel_miR2745	5p	0	198.2	0	0	AACUGGUCUUGCUUAAUUUUG	21	AGAUUAAGCAAGGUCACUUGA	21
novel_miR2917	3p	0	5.5	0	0	AUGCCGUGUUGUUCUGAAAGAAA	23	UCUUCAGAAGAGCAUGUCAUUU	22
novel_miR3314	5p	0	6.7	0	0	UGAAGAGCGCGGGCAGCACAA	21	GUGCUGCCCGCGCUCUUCAUG	21
novel_miR3417	3p	0	0.2	0.3	2.8	GAAUGGGAUUCGCUUGGGCAA	21	UUCCAACGGACUCAUUCCA	19
novel_miR3607	3p	0	1.0	0	13.8	GAAUGGGAUUCGCUUGGGCA	20	UCCAACGGACUCAUUCCAUG	20
novel_miR3791	3p	0	0	1.2	12.6	GAGAGGGAGGGCAGAGGCCU	20	AAGCUCUGCCCUUCCAUCUCUUG	23
novel_miR4191	5p	0	0	11.5	0	CACGAUACUCUUGGACGAUUC	21	AUCGUCCAGCAGUAUCGUCUG	21
novel_miR4740	3p	0	0	7.3	0	AAAAAAGAUUGAGCCGAAUAA	21	ACUCGGCUCAAUCUUUUUUUU	21
novel_miR5134	3p	0	0	0	5.7	UGGAUACCGCCCAACUCUGUU	21	CAGAGUUGGGCAGUAUCCAUG	21
novel_miR5169	5p	0	0	0	88.6	UAAUCUUCUGGAUAUAUGCUUG	22	AGUAUGUUUUUAGAAGAUUAGA	22
novel_miR5172	3p	0	0	0	4.8	GGCGGCAUGGGGAUGUCAAAA	21	UUGGCGUCCCCAUGUCCCCC	20
novel_miR5542	5p	0	0	0	28.6	AUGAAGAGCGCGGGCAGCACA	21	UGCUGCCCGCGCUCUUCAUGG	21
novel_miR5552	5p	0	0	0	3.0	CCUCCCGGACCAGAACUUCUU	21	GAGGUUUUGGUCAGGGGGACG	21
novel_miR5598	3p	0	0	0	2.7	GAGCACGGGCUUUUCUGGUCA	21	ACCGAAGAAGCCUGUGCUCGA	21
novel_miR5756	3p	0	0	0	10.2	UAGAGAGGGAGGGCAGAGGC	20	CUCUGCCCUUCCAUCUCUUGG	21
novel_miR5816	5p	0	0	0	4.9	GGUCUGUGUUUGUUUCAAAC	20	UUGAGACGAAAACAGACCAA	20
novel_miR5831	3p	0	0	0	2.6	UCGAGAUCCAACGGCUGAGGU	21	CUCGGCCGUUGGAUCUUUGAUA	22

TPM, tags per million reads; target for novel_miR692-5p was validated by RLM-5’RACE.

### Identification of Differentially Expressed miRNAs

A knowledge of the expression patterns of differentially expressed miRNAs during cold acclimation and vegetative/reproductive transition of winter wheat could provide clues about their potential functions. We first compared the expression patterns of miRNAs in leaves at different growth and developmental stages. All the known miRNAs were clustered by subjecting them to *K*-means clustering using Euclidian distance measures with Genesis v1.8.1 (Sturn et al., 2002). The results revealed several distinct expression patterns (**Supplemental Figure S1**). Most miRNAs had similar expression patterns at the four growth stages; the expression level was either high (clusters 7 and 10) or low (clusters 5 and 11). miRNAs in clusters 1 and 8 were mainly expressed at the jointing stage, whereas miRNAs in clusters 13 and 14 were highly expressed at seedling stages, and their expression was extremely suppressed at the jointing stage; miRNAs in cluster 15 were relatively induced at the spring green-up and jointing stages. miRNAs in cluster 16 were induced at the spring green-up stage. miRNAs in cluster 9 were highly expressed in the cold winter and jointing stages.

**Figure 2:**
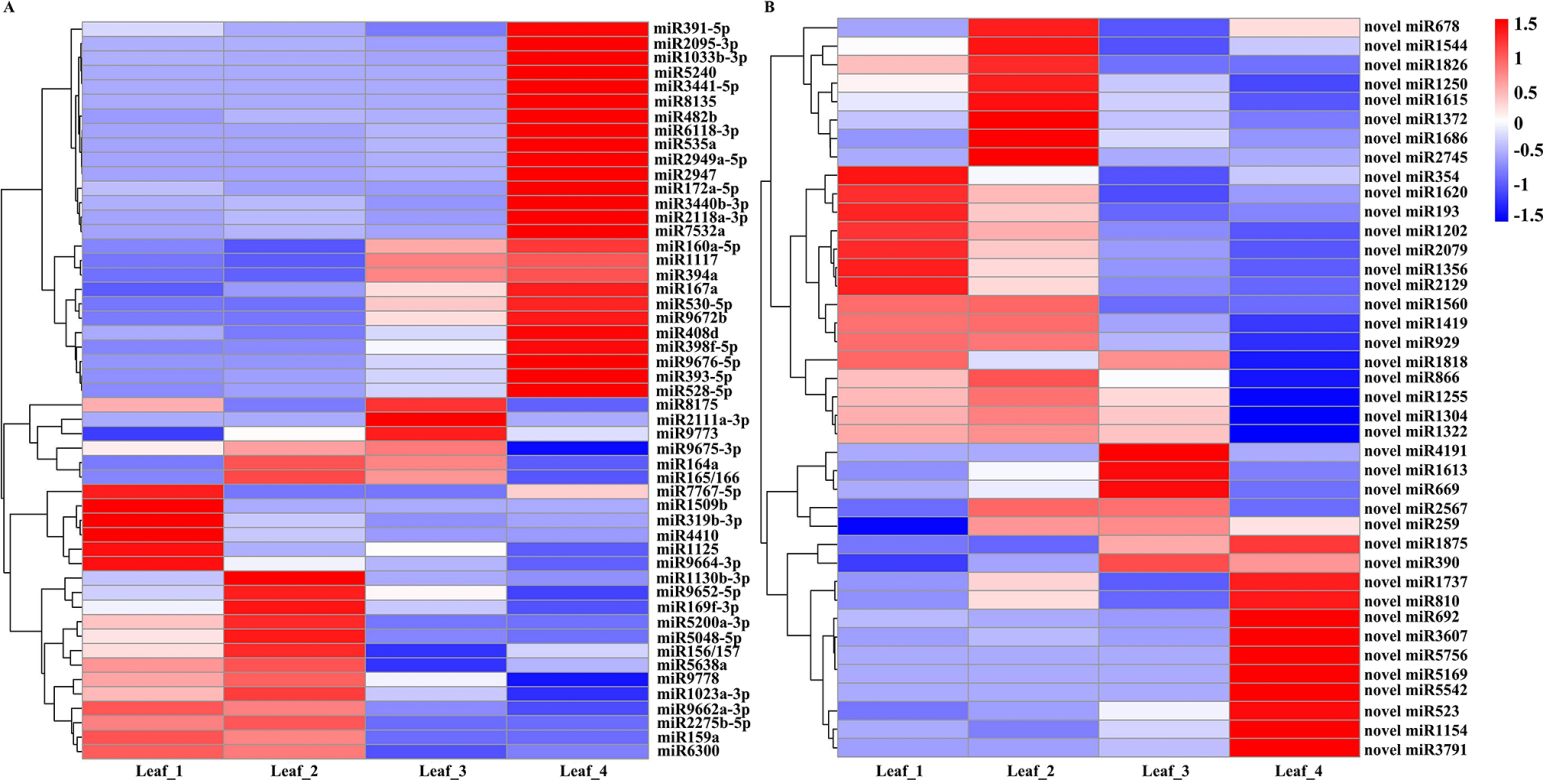
Heat map of differentially expressed known miRNAs **(A)** and novel miRNAs **(B)** in wheat leaves at four different development stages. Each column represents a stage, and each row represents a miRNA. The bar represents the scale of relative expression levels of miRNAs.

We further selected DEG miRNAs by considering fold changes greater than twofold between any two stages (|log2 fold change| > 1, *p*-value < 0.01) and with more than 5 TPM in at least one stage. A total of 51 known miRNAs were designated as significantly differentially expressed miRNAs. Generally, 19 miRNAs, including miR159a, miR1125, miR169f-3p, miR319b-3p, miR1130b-3p, miR5200a-3p, and miR9662a-3p, were abundant in at least one of the vegetative stages and had low expression levels at the spring green-up and jointing stages. miR8175, miR2111a-3p, and miR9773 were significantly induced at the spring green-up stage. miR164a, miR165/166, and miR9675-3p were highly expressed at the winter dormancy and spring green-up stages. Six miRNAs (miR160a-5p, miR167a, miR394a, miR530-5p, miR1117, and miR9672b) were induced at the spring green-up stage and exhibited the highest expression level at the inflorescence initiation stage; 20 miRNAs, including miR391-5p, miR172a-5p, miR393-5p, miR398f-5p, miR408d, miR528-5p, miR2095-3p, miR5240, miR2118a-3p, miR7532a, and miR9672b, were highly induced at the jointing stage (**Figure 2A**). Forty novel/putative miRNAs were identified as DEG miRNAs. Ten novel miRNAs (novel_miR354, novel_miR1620, novel_miR193, novel_miR1202, novel_miR2079, novel_miR1356, novel_miR2129, novel_miR1560, novel_miR1419, and novel_miR929) showed higher expression at the three-leaf and winter dormancy stages; five novel miRNAs (novel_miR1818, novel_miR866, novel_miR1255, novel_miR1304, and novel_miR1322) were abundantly expressed at the three-leaf stage, winter dormancy stage, and spring green-up stage, while they were suppressed at the jointing stage. Novel_miR2567 and novel_miR259 were preferentially expressed at the winter dormancy and spring green-up stages; novel_miR4191, novel_miR1613, and novel_miR669 were highly expressed at the spring green-up stage but were suppressed at the jointing stage; novel_miR1875 and novel_miR390 were induced at the spring green-up and jointing stages. Ten novel miRNAs (novel_miR1737, novel_miR810, novel_miR692, novel_miR3607, novel_miR5756, novel_miR5169, novel_miR5542, novel_miR523, novel_miR1154, and novel_miR3791) were exclusively induced in leaves at the jointing stage (**Figure 2B**).

Under natural growth conditions, winter wheat is simultaneously exposed to cold weather and short photoperiod conditions in winter and then transits from the vegetative to reproductive growth in the next spring. Comparison of miRNA expression at winter dormancy and the three-leaf stage might reveal miRNAs essential for cold acclimation and/or miRNAs responsive to a short photoperiod. Compared to the three-leaf stage, miR7767-5p, miR1509b, miR319b-3p, miR1125, miR4410, miR9664-3p, and eight novel miRNAs (novel_miR354, novel_miR1620, novel_miR193, novel_miR1202, novel_miR2079, novel_miR1356, novel_miR2129, and novel_miR1818) in leaves were suppressed at the winter dormancy stage, whereas miR164a, miR165/166, miR1130b-3p, miR169f-3p, miR5048-5p, miR5200a-3p, miR9652-5p, and eight novel miRNAs (novel_miR678, novel_miR1544, novel_miR1826, novel_miR1250, novel_miR1615, novel_miR1372, novel_miR1686, and novel_miR2745) were significantly induced in winter and were suppressed at the spring green-up stage (**Figure 2**). These data specify the potential regulatory function of miRNAs in modulating the growth and development of wheat at the winter dormancy stage.

**Figure 3:**
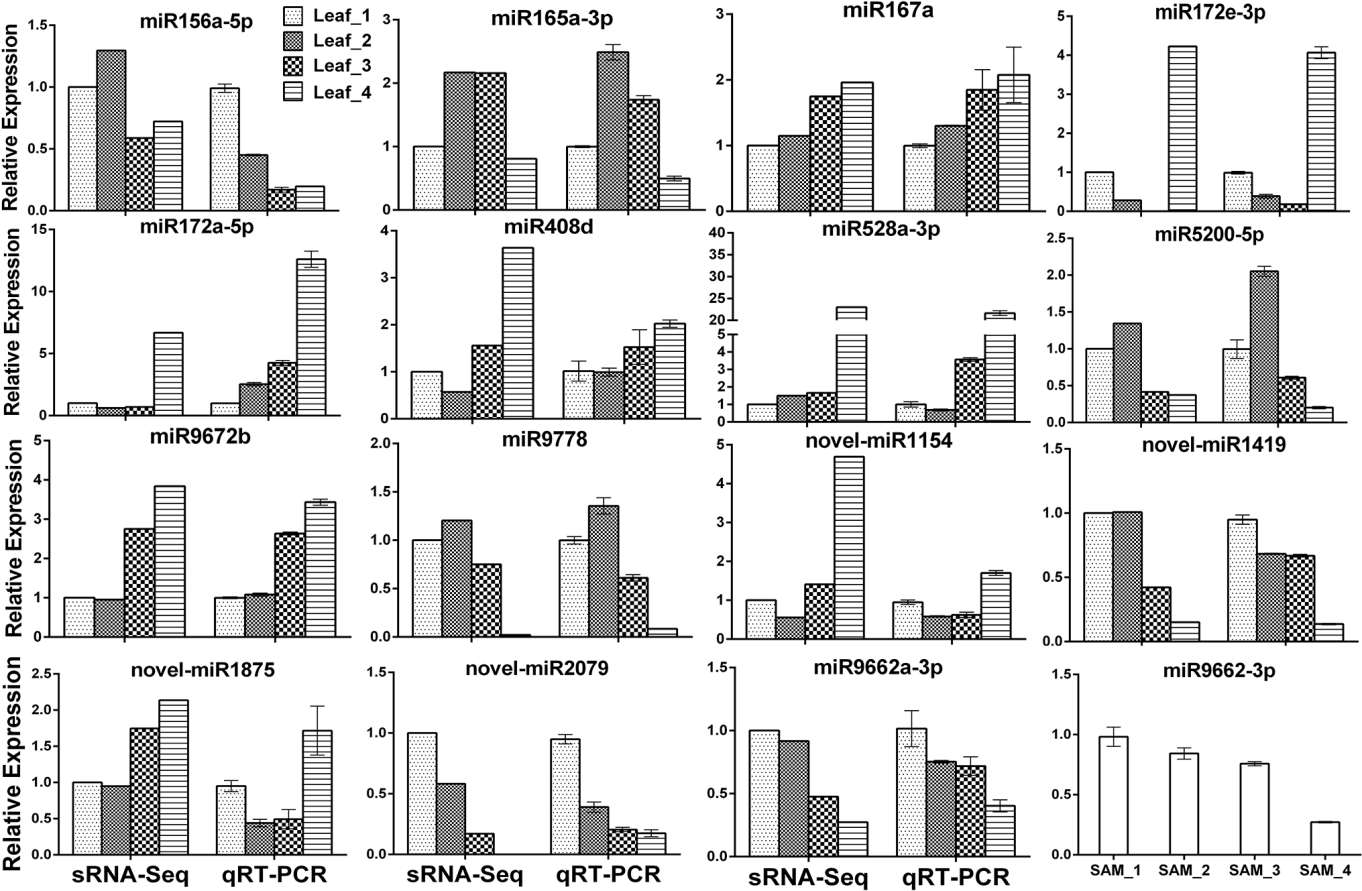
Validation of differentially expressed known and novel miRNAs derived from high-throughput sequencing by qRT-PCR.

To validate the expression profiles obtained by sRNA-Seq, the RNA samples were first polyadenylated and then reverse-transcribed to cDNA. Eighteen DEG miRNAs were randomly selected for verification by qRT-PCR; three of them failed due to multiple amplification products. The expression patterns of the remaining 15 miRNAs detected by qRT-PCR were consistent or partially consistent with those determined by deep sequencing, although the fold change in expression varied between the sRNA-Seq and qRT-PCR results (**Figure 3**). Previous studies have shown that miR156 is highly expressed in juvenile plants and is suppressed in adult plants. Compared with the three-leaf stage, the expression of miR156a-5p at the spring green-up stage and jointing stage was relatively low by both sRNA-Seq and qRT-PCR, while the downregulation pattern at the winter dormancy stage detected by qRT-PCR was not observed by sRNA-Seq (**Figure 3**), probably due to the two mismatches allowed during the annotation of known miRNAs.

### Prediction of Potential Target Genes of Known and Novel miRNAs

Identification of miRNA target transcripts is essential to comprehensively understand miRNA-mediated gene regulation. Potential target transcripts for the known and novel miRNAs from wheat were predicted using the PsRobot pipeline (only those with alignment scores between the query miRNA and targets less than 2.5 were considered). Consequently, 4,780 targets for 224 known miRNAs and 1,467 targets for 48 novel miRNAs and 25 putative miRNAs were predicted (**Supplemental Tables S7**–**S8**). A number of targets were found to be TFs, including MYB TFs (target of miR159), homeobox-leucine zipper protein (target of miR164), TF IIIB 90 kDa subunit (target of miR394), and MADS-box TF (target of miR444). Other conserved miRNA targets, including transport inhibitor response 1 (target of miR393), auxin response factor (target of miR160 and miR167), sulfate transporter 2 (target of miR395), laccase (target of miR397), and Cu/Zn superoxide dismutase (SOD, target of miR398) were also found (**Supplemental Table S7**). In addition to the well-documented conserved targets, three *VRN3* genes were also predicted as targets of miR5200 (**Supplemental Table S7**). Many novel targets were also predicted for known and novel miRNAs. Although most of the predicted miRNA–target relationships need to be further validated experimentally, these results strongly suggest that wheat miRNAs are involved in the regulation of various biological processes, like plant architecture, development, and biotic and abiotic stress response.

**Figure 4:**
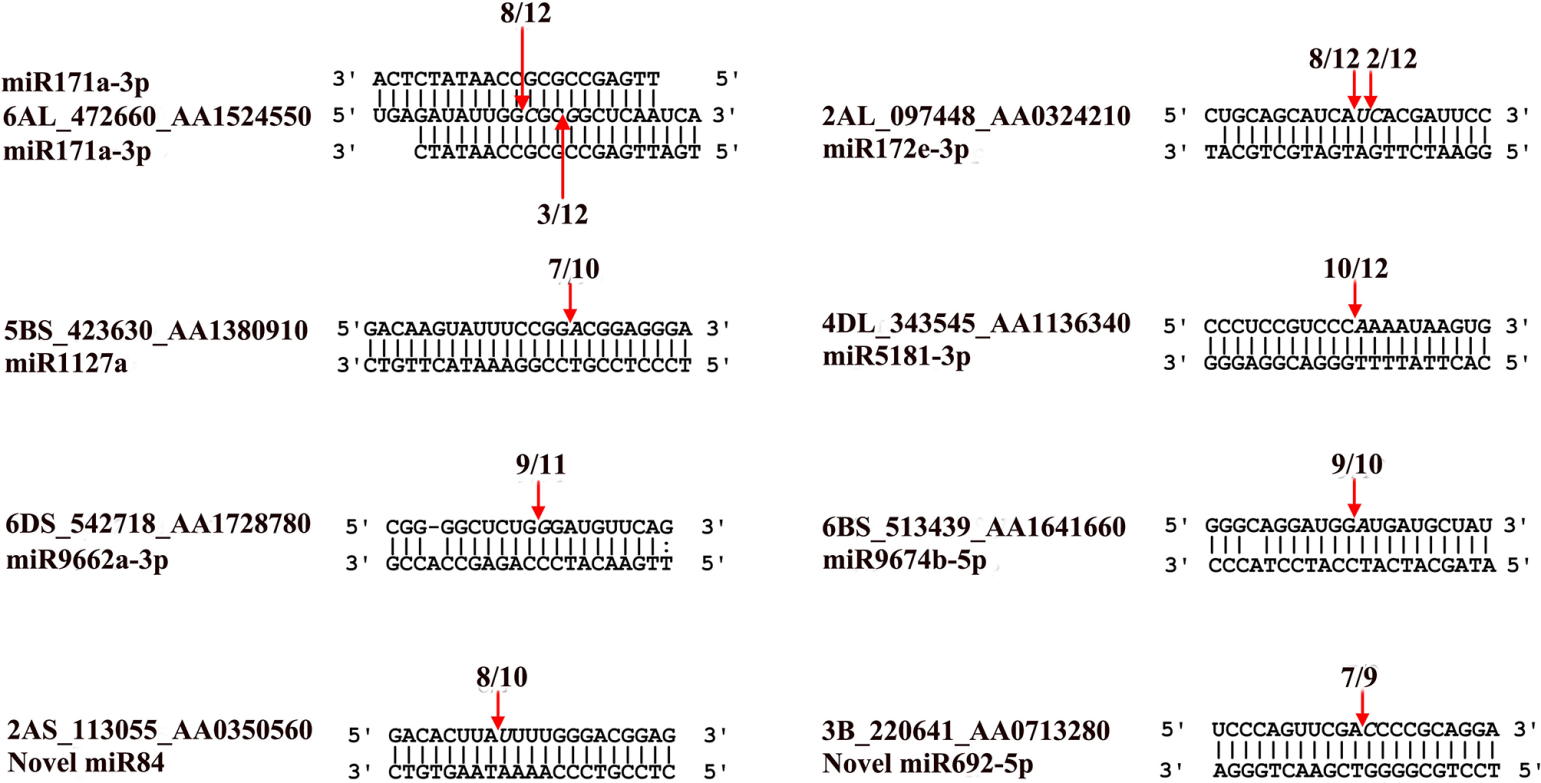
Validation of known and novel miRNA targets using RLM-5’RACE assay. Arrows indicate the cleavage sites detected by RLM-5’RACE.

Using the RLM-5′RACE technique, eight genes were verified as *bona fide* miRNA targets (**Table 3** and **Figure 4**). Among them, two conserved targets for miR171 (TRIAE_CS42_6AL_TGACv1_472660_AA1524550.1, scarecrow-like protein) and miR172 (TRIAE_CS42_2AL_TGACv1_097448_AA0324210.1, AP2-like ethylene-responsive TF) were verified. Four novel targets for *Pooideae*-specific/wheat-specific miRNAs were validated. miR1127 was found only in wheat and *Brachypodium distachyon*; we confirmed that this miRNA targets TRIAE_CS42_5BS_TGACv1_423630_AA1380910, a disease resistance protein, RGA1. miR5181, miR9662a-3p, and miRNA9674 were found in wheat, and its progenitor, *Aegilops tauschii*, TRIAE_CS42_4DL_TGACv1_343545_AA1136340 (a predicted protein), TRIAE_CS42_6DS_TGACv1_542718_AA1728780 (transcription termination factor mTERF15), and TRIAE_CS42_6BS_TGACv1_513439_AA1641660 (protein Rf1, mitochondrial-like) were verified as their corresponding targets. Notably, targets for two novel miRNAs were also verified. TRIAE_CS42_3B_TGACv1_220641_AA0713280 (integrator complex subunit 9) and TRIAE_CS42_2AS_TGACv1_113055_AA0350560 (beta-galactoside alpha-2, 3-sialyltransferase) were targeted by novel_miR692-5p and novel_miR84, respectively. It is worth noting that no miRNA* was detected for novel_miR84, but its precursor has a perfect fold-back structure (**Supplemental File S2**).

**Table 3:** Validated miRNA target genes and primer sequence.

miRNA	Target gene ID	Primer sequence (5’–3’)	Target annotation
novel_miR692-5p	3B_220641_AA0713280.1 (Traes_3B_E1131EA03.1)	Rev1: AACACCAATCTCCGTCCGTC	Integrator complex subunit 9 homolog
		Rev2: AGTAGAATCGATCGCTACAAC	
novel_miR84	2AS_113055_AA0350560.2 (Traes_2AS_6BF19AB61.1)	Rev1: ACAGACCGTATGCTAGCAATG	Beta-galactoside alpha-2,3-sialyltransferase
		Rev2: ACACTCCTGTGTTCAGCCTC	
tae-miR1127a	5BS_423630_AA1380910.2	Rev1: ACTGCACGATATTTGGATGCTTAAC	Putative disease resistance protein RGA1
		Rev2: TATTCTTGAGTCCACTGAGAATCC	
tae-miR171a-3p	6AL_472660_AA1524550.1 (Traes_6DL_26DDCA106.1)	Rev1: AGTACCACGGCGATGAGCAAG	Scarecrow-like protein 6
		Rev2: AGGCGATTTCGTCGAGAAGC	
tae-miR172e-3p	2AL_097448_AA0324210.1 (Traes_2AL_5BA7E2623.1)	Rev1: ACCACATCCATCCGTCCTCTC	AP2-like ethylene-responsive TF
		Rev2: AGTTCCAGTTGAGTTGTGGTG	
tae-miR5181-3p	4DL_343545_AA1136340.2 (Traes_4DL_9294C72F2.1)	Rev1: AGCGAGTACACATATACAGGACAG	*vulgare* mRNA for predicted protein
		Rev2: TAACAGGAGGCAAAATGCCG	
tae-miR9662a-3p	6DS_542718_AA1728780.1 (Traes_6DS_F6C7E588F.1)	Rev1: AGCTCAAGTCACGATCTAGCAG	Transcription termination factor MTERF15
		Rev2: TGATCGATGAGCAATGTACACG	
tae-miR9674b-5p	6BS_513439_AA1641660.1	Rev1: TTACCTTCTGCAGCCTATGAC	Protein Rf1, mitochondrial-like
		Rev2: AGCTCTGTCATGAGGTACCCTG	

3B_220641_AA0713280.1 is a short name of TRIAE_CS42_3B_TGACv1_220641_AA0713280.1 in ftp://ftp.ensemblgenomes.org/pub/plants/release-37/fasta/triticum_aestivum/; the same abbreviation applies to other target gene IDs. Gene ID in parenthood is the gene ID in  ftp://ftp.ensemblgenomes.org/pub/plants/release-31/fasta/triticum_aestivum/.

## Discussion

Winter wheat requires prolonged exposure to cold temperatures for acceleration of flowering. miRNAs play important roles in the growth and development of plants, adjusting to various stress and hormone response (Rogers and Chen, 2013; Yu et al., 2017). To investigate the potential roles of miRNAs during cold acclimation and vegetative/reproductive transition of winter wheat under natural growth conditions, we performed a comprehensive analysis of sRNAs in the wheat leaves at four different growth and development stages. In total, 373 known miRNAs belonging to 289 miRNA families, 55 novel miRNAs, and 27 putative novel miRNAs were identified by high-throughput sequencing. The abundance of conserved miRNAs varied; miR168 was found to be the most abundant conserved miRNA. Other highly expressed miRNAs included miR159, miR167, miR156, miR66, miR167, miR5048, miR5062, miR7757, miR9662, miR9672, and miR9674, in agreement with the results of a previous report (Han et al., 2014). Conserved miRNAs and their corresponding target genes are commonly found in all or most of the plant species (Pokoo et al., 2018). All 23 most conserved miRNAs (miR156/157, miR159, miR160, miR161, miR162, miR164, miR165/166, miR167, miR168, miR169, miR170/171, miR172, miR319, miR390, miR391, miR393, miR394, miR395, miR396, miR397, miR398, miR399, and miR408) in eudicots (Zhang et al., 2011; Li et al., 2013; Meng et al., 2013; Pokoo et al., 2018) and miR444, a monocot-specific miRNA (Sunkar et al., 2008), were identified in this study, suggesting that these highly conserved miRNA families are evolutionarily conserved across all major lineages of plants and play specific functions in a variety of physiological processes in plant species. The number of less conserved and wheat-specific miRNAs is very high; this fact, together with the diverse expression pattern of miRNAs at four different growth and development stages (**Figure 2**, **Supplemental Tables S2**, **S4**, and **S5**), indicates the wide range of regulatory functions mediated by miRNAs in leaves during wheat growth and development.

MicroRNAs function by regulating target genes *via* sequence complementary based cleavage or translation repression (Llave et al., 2002; Rogers and Chen, 2013; Yu et al., 2017). Therefore, identification of miRNA target transcripts is essential for comprehensive understanding of miRNA-mediated gene regulation. Bioinformatics analysis predicted 4,780 targets for 224 known miRNAs (**Supplemental Table S7**) and 1,467 targets for novel and putative novel miRNAs (**Supplemental Table S8**). A number of conserved target genes were identified, including MYB TFs, homeobox-leucine zipper protein, MADS-box TF, transport inhibitor response 1, auxin response factor, sulfate transporter 2, laccase, and Cu/Zn SOD (**Supplemental Table S7**). Besides these known targets, a large number of novel targets for known miRNA and novel miRNAs were also predicted (**Supplemental Tables S7**–**S8**). Because not all predicted targets are the real targets, experimental validation is necessary to illustrate the biological function of these miRNAs in wheat. Using RLM-5′RACE, we confirmed eight miRNA target genes. Among these, two genes are conserved targets for miR171 and miR172, respectively; four targets for four *Pooideae*-specific/wheat-specific miRNAs were validated. miR1172a targets RGA1, a disease resistance protein; miR5181-3p targets a predicted protein; miR9674b-5p targets Rf1; TRIAE_CS42_6DS_TGACv1_542718_AA1728780.1, encoding mTERF15, is a target gene of miR9662a-3p (**Figure 4**). The mTERF family is comprised of a wide range of proteins localized in mitochondria, which have been shown in *in vitro* and *in vivo* studies to have roles in transcription initiation and DNA replication in animals, in addition to transcription termination (Roberti et al., 2009). Plant mTERFs also localize to chloroplast. A previous study showed that mTERF6 is required for maturation of chloroplast tRNA_ILE_ in *Arabidopsis* (Romani et al., 2015). Mutations in *mTERFs* alter the expression of organellar genes and impair the development of chloroplast and mitochondria. Changes in nuclear gene expression were also observed in *mterf* plants (Quesada, 2016). Moreover, a recent study revealed that mTERF5 can cause plastid-encoded RNA polymerase (PEP) complex to pause at *psbEFLJ* (encoding four key subunits of photosystem II) in *Arabidopsis*, confirming that RNA polymerase transcriptional pausing also occurs in plant organelles (Ding et al., 2019) and indicating the critical role of mTERFs in the growth and development of plants. We found that the expression of miR9662a-3p in leaves was gradually decreased with the growth of wheat plants (**Figure 3**) and speculate that it might be an important regulator during wheat growth and development. We further checked the expression of miR9662a-3p in shoot apical meristem (SAM) at the same growth stage and found a similar expression pattern in SAM to that in leaves (**Figure 3**). Further studies are required to determine the potential roles of mTERF15 in wheat. Most importantly, targets for two novel miRNAs were validated in this study; novel_miR692-5p targets an integrator complex subunit 9, and novel_miR84 targets beta-galactoside alpha-2,3-sialyltransferase. These results not only prove that they are true miRNAs but also demonstrate that these novel miRNAs are functional in modulating the expression of their target genes at the post-transcriptional level in wheat.

A total of 91 differentially expressed miRNAs were identified. Conserved miR156, miR164a, miR165/166, miR159a, miR169f-3p, and miR319b-3p were highly expressed at the vegetative growth stage, and miR160a-5p, miR167a, miR172a-5p, miR393-5p, miR398f-5p, and miR408d were abundantly expressed at the jointing stage (**Supplemental Table S2** and **Figure 3**). The target genes of the most conserved miRNAs are TFs (Li et al., 2010). It is well known that a TF can regulate the expression of multiple downstream target genes by either activation or suppression; therefore, the different regulation patterns of these conserved miRNAs imply that complicated transcription and post-transcription regulation underlie the growth and development processes in winter wheat. Previous studies have shown that miR156 and miR172 have opposite patterns of temporal regulation in several plant species (Wu and Poethig, 2006; Poethig, 2009; Wu et al., 2009; Gao et al., 2017). In this study, the expression level of miR156a-5p in leaves was low at the spring green-up stage and jointing stage compared with that at the three-leaf stage, while miR172 was expressed at very low levels during the vegetative growth and spring green-up stages, but its expression was increased significantly at the jointing stage (**Figure 3**), confirming that the regulatory circuit of the miR156/miR172 module is highly conserved among phylogenetically distinct plant species and plays important roles in regulating flowering (Wang and Wang, 2015; Wang et al., 2015). miR156 was reported to be induced by cold stress in *Brassica rapa* (Zeng et al., 2018), whereas it was downregulated by cold stress in rice (Cui et al., 2015), young spikes of wheat (Song et al., 2017), and *Astragalus membranaceus* (Abla et al., 2019). Overexpression of rice miR156 in *Arabidopsis*, pine, and rice resulted in improved cold stress tolerance by increasing cell viability and growth rate under cold stress (Zhou and Tang, 2018). The altered expression of miR156a-5p at the winter dormancy stage indicates that miR156 is not only a flowering regulator but also a cold stress responder in plants.

MicroRNAs have been demonstrated to be involved in the regulation of cold stress in different plant species (Megha et al., 2018). In the present study, the expression of miR319b-3p, miR1125, miR1509, miR4410, miR7767, and miR9664 was highly repressed at the winter dormancy stage compared to their expression at the three-leaf stage (**Figure 2A**, **Supplemental Table 2**). In previous studies, it has been reported that miR319 is a cold stress–responsive miRNA, which was shown to be upregulated in *Arabidopsis* and sugarcane (Sunkar and Zhu, 2004; Thiebaut et al., 2012) but was downregulated by cold stress in rice (Yang et al., 2013; Wang et al., 2014b). Overexpression of miR319 or RNA interference of its targets (*OsPCF5* RNAi and *OsPCF8* RNAi) in rice leads to the upregulation of cold-responsive genes and improved cold tolerance (Yang et al., 2013; Wang et al., 2014b). The downregulation of miR319b-3p at the winter dormancy stage demonstrated the essential role of miR319 in cold acclimation of wheat grown in the field. Compared to the three-leaf stage, the expression of miR164a, miR165/166, miR169f-3p, miR1130b-3p, miR5048-5p, miR5200a-3p, and miR9652-5p was significantly induced in winter (**Figure 2**). The induction of miR164 and miR169 by cold stress has been reported in *Astragalus membranaceus* (Abla et al., 2019); miR165 and miR1130 were reported to be induced by cold stress in young wheat spikes (Song et al., 2017). Wheat miR166 was induced at the winter dormancy stage, in agreement with the previous finding that it is a cold-responsive miRNA in *Brassica rapa* (Zeng et al., 2018).

*FLOWERING LOCUS T* (*FT*) is a key gene involved in the regulation of flowering time. FT is a mobile florigen, transported from leaves into the phloem and then to SAMs, and initiates phase transition (Corbesier et al., 2007). Wheat *FT1 (VRN3)* is one of the most important flowering promoters. Expression of *VRN3* is activated by vernalization, suppressed in short-day conditions, and induced under long-day conditions (Yan et al., 2006). Recent studies revealed that expression of *VRN3* is also directly or indirectly regulated by miRNAs at the post-transcription level. miR5200, a *Pooideae*-specific miRNA, targets *FT* orthologs in *Brachypodium distachyon*. bdi-miR5200 was reported to be abundantly expressed in the leaves of plants grown under short-day conditions but was dramatically suppressed in plants under long-day conditions (Wu et al., 2013). It was reported that wheat miR5200 is under negative control of the light receptor PhyC and also targets *VRN3* in wheat (Pearce et al., 2016). We found that wheat miR5200a-3p was highly expressed in leaves under short-day conditions (three-leaf stage and winter dormancy stage), indicating that miR5200 functions in the degradation of *VRN3* transcripts and provides another layer of assurance that flowering does not occur in late autumn or transient warm conditions in winter. Low expression level of miR5200a-3p at the spring green-up and jointing stages contributes to the accumulation of *VRN3* transcript and protein. TIME OF CAB EXPRESSION 1 (TOC1) is a key component of the plant circadian clock and is closely linked to the CO-FT flowering pathway (Niwa et al., 2007). miR408 is a copper deprivation responsive miRNA and targets plantacyanin and copper-containing protein genes (Li et al., 2010; Zhang et al., 2011). Wheat *TOC1s* were confirmed as target genes of miR408; both knockdown of wheat *TOC1* and overexpression of miR408 resulted in early heading phenotype (Zhao et al., 2016); further study revealed that miR408-mediated cleavage of *TOC1s* could activate the expression of wheat *VRN3* gene and promoted phase transition (Zhao et al., 2016). sRNA-Seq showed that the expression level of miR408 was relatively low at all four stages, but induction of miR408d at the jointing stage was obvious and was validated by qRT-PCR (**Figure 3**), confirming previous findings that miR408 is a promoter of wheat flowering.

miRNA159 targets GAMYB TFs. Previous studies in wheat, barley, and rice identified the role of GAMYB in anther development (Murray et al., 2003; Kaneko et al., 2004; Wang et al., 2012b). We found that the expression of wheat miR159a was significantly suppressed at the spring green-up stage and jointing stage (**Figure 2** and **Supplemental Table S2**), which suggests that miR159 is a suppressor of flowering in wheat. This correlates well with a previous finding that elevated expression of miR159 results in delayed flowering in *Arabidopsis* (Achard et al., 2004). miR2111a-3p, miR8175, and miR9733 were significantly induced in wheat leaves at the spring green-up stage. The induction of miR2111 after cold stress was reported in *A. membranaceus* (Abla et al., 2019). Auxin efflux carrier and mitogen-activated protein kinase kinase kinase 13 were predicted as targets of miR2111 (**Supplemental Table S7**); however, the exact biological function of miR2111 in regulating growth and development in wheat remains unclear.

Several miRNAs, such as miR160a-5p, miR167a, miR172a-5p, miR391-5p, miR394a, miR398f-5p, miR528-5p, and miR530-5p, were highly expressed at the jointing stage. miR528 is a monocot-specific miRNA, which accumulates in old plants of rice and exhibits diurnal rhythms of expression. It promotes flowering in rice under long-day conditions by targeting *RED AND FAR-RED INSENSITIVE 2* (*OsRFI2*) (Yang et al., 2019). In wheat, the expression of miR528-5p was significantly induced at the jointing stage (**Figure 2A**), indicating that miR528-5p is essential for wheat flowering. In a previous study, it was shown that miR167 was abundantly accumulated in floral organs during the early stage of anther development in rice (Fujioka et al., 2008). In this study, miR167a was observed to be constantly induced after the winter season, and its expression reached the highest level at the jointing stage (**Figure 2A**, **Supplement Table S1**), indicating that miR167 might function as a promoter of flowering in wheat.

*Cu/Zn SODs* are target genes of miR398. miR398 is expressed at optimal levels under normal growth conditions and is downregulated by oxidative stress in *Arabidopsis* (Sunkar et al., 2006). The inverse relationship between miR398 and *Cu/Zn* *SODs* has been observed under cold stress in wheat and other plants (Lu et al., 2010; Wang et al., 2014a). Expression of miR398 in wheat leaves was not altered at the winter dormancy stage. Other cold stress–responsive miRNAs, such as miR394, miR396, and miR397 (Megha et al., 2018), did not show significant changes between the three-leaf stage and winter dormancy stage, the main reason for which could be attributed to the fact that winter wheat was grown in the field under natural weather conditions in this study. The temperature was fluctuant and decreased gradually, and the duration of cold weather was very long (cold acclimation), while most cold stress studies were conducted in controlled environments for short periods (cold stress). Moreover, photoperiod is another parameter; the impact of photoperiod on the expression of miRNAs has been confirmed (Wu et al., 2013; Pearce et al., 2016). Most of the studies on cold stress–responsive miRNAs were done under certain photoperiod conditions, while the photoperiod varied at different stages in this study.

Besides these conserved miRNAs, 30 other less conserved known miRNAs and 40 novel miRNAs were found to be differentially expressed in leaves during cold acclimation and vegetative/reproductive transition of winter wheat. Although target genes for most of these miRNAs were predicted, their regulatory role in the growth and development of wheat can only be clarified upon further experimental validation.

## Conclusions

We performed a transcriptome-wide identification and characterization of miRNAs from wheat leaves at four different stages of growth and development. A total of 373 known miRNAs belonging to 289 miRNA families and 82 novel miRNAs were identified. Ninety-one miRNAs were found to be differentially expressed at the four stages. The expression of six known and eight novel miRNAs was suppressed at the winter dormancy stage, whereas seven known and eight novel miRNAs were induced at this stage. Twenty known miRNAs and 10 novel miRNAs were significantly induced at the jointing stage. The expression of a number of representative miRNAs was verified by qRT-PCR analysis. Eight miRNA target genes were validated using an RLM-5′RACE assay; these included targets for two novel miRNAs and four *Pooideae*-specific miRNAs. This study provides valuable information for in-depth understanding of the regulatory mechanism of miRNAs in the growth and phase transition of winter wheat. Further studies are required to decipher how these miRNAs are involved in the growth and development of wheat.

## Data Availability

The sequencing data of this study have been deposited at the National Center for Biotechnology Information Gene Expression Omnibus (NCBI, GEO, http://www.ncbi.nlm.gov/geo/) under accession number GSE125391.

## Author Contributions

Y-FL conceived the idea and designed the study. KW, MW, LW, and DZ performed sample collection and RNA extractions. Y-FL, KW, MW, JG, and YZ analyzed the sRNA library data sets. KW, MW, JC, and MZ performed qRT-PCR analyses and miRNA target validation. Y-FL and KW prepared the manuscript. All authors reviewed and approved the final manuscript.

## Funding

This study was supported by grants from the National Natural Science Foundation of China (no. 31771703) and a stand-up fund from Henan Normal University to Y-FL.

## Conflict of Interest Statement

The authors declare that the research was conducted in the absence of any commercial or financial relationships that could be construed as a potential conflict of interest.
