# Supplementary material for: Identification and Temporal Expression Analysis of Conserved and Novel MicroRNAs in the Leaves of Winter Wheat Grown in the Field
Source: Front Genet. 2019 Sep 4;10:779. doi: 10.3389/fgene.2019.00779 (PMC6737308; doi:10.3389/fgene.2019.00779)
Supplement: Supplementary file 9 [file Table_9.docx]

**Supplemental file 1. Foldback structure of novel MIRNA (with miRNA*) predicted by mfold server**

1. ***novel_MIRNA*_30**

10 20 30 40 50 60

CUUU GA- U AUCAAC C .-GCAAAAUCAGCUUCAC| A

AAGUUUGGCCAAGUCUA GAA AAUGU AGC ACAGC CAU A

UUUAAACUGGUUCAGAU CUU UUACA UUG UGUUG GUA A

UUUC AUC - CGAUUA A \ ----------------^ A

. 140 130 120 110 70

80 90

UACUU U A

UCG AAUAUAU U

AGU UUAUAUA A

U---- - U

100

1. ***novel_MIRNA_127***

10 20 30 40 50 60

- - A C A .-CUUGAA| A GA

CUG CUUCUCAUUUGAAGACU GUUUAUUA AU UAAGCAUAUU UGUUUG GA G

GAC GAAGAGUAGAUUUCUGA CAAAUAAU UA AUUUGUAUAA ACGAAC CU A

A G A A G \ ------^ A AG

190 180 170 160 150 70

80 90 100

GA U .-A A-- C A C

UCUCG GC GAAG GAG AUCAUUG UA A

AGAGU CG CUUC UUC UAGUAAU AU A

-- - \ - ACC U - C

120 110

130

AAGA A

GCC U

1. ***novel_MIRNA_128***

10 20 30 40 50 60

- - U A C A UU .-AGA| ACA

CUG CUUCUCAUC GAAGACU GUUUAUUA AU UAAGCAU--GUUUUUGGAAGU GAGAG GAUC \

GAU GAAGAGUAG UUUCUGA CAGAUAAU UA AUUUGUA CGAGAACCUUCA UUCUU CUAG A

A G U A A G \ CC \ ---^ ACG

190 180 170 160 130 120

80 90

CC--------- A A GA

GUGU GA GA \

CAUA UU CU A

AAUAAUAUCAA G A AC

110 100

140

CA--- UG A

U G C

A C C

UAAAG GU A

150

1. ***novel_MIRNA_193***

10 20 30 40 50 60 70 80 90

U G A -| A A G C C AG G G A

GUACAAUG GAG UGCUUA GGGAGGUGUUUAGA AAAUAAACCG GUUUUUCU AAGCACCGGUGC UAUUU UAU GA AGA GCUU G

CAUGUUAC UUC ACGAAU CCCUCCACGAAUCU UUUAUUUGGU CGAAAGGA UUCGUGGCCAUG AUAAA AUG CU UCU CGAA U

- G C C^ C C A A C GA A G U

180 170 160 150 140 130 120 110 100

1. ***novel_MIRNA_259***

10 20 30 40

-| C A A G U GG - CU

GUG UCAGG GAG UGACACCGAC CCGA CAGAU GUC GG \

UAC AGUCC CUC ACUGUGGUUG GGCU GUCUG CGG CC U

A^ A G C A U -- A AA

80 70 60 50

1. ***novel_MIRNA_364***

10 20 30

C| G

GAUACUGGUUCAGGACGUUGCAACAUUAACACCU U

CUAUGACCAAGUCCUGCAACGUUGUAAUUGUGGA U

A^ C

70 60 50 40

1. ***novel_MIRNA_381***

10 20 30 40

-| G G A UGUAU

UCCGUUC GAAUUACUUGUC CG AAAUGGAUGUAUCUAGA \

AGGCAAG UUUAAUGAACAG GC UUUACCUACAUAGAUCU U

G^ G A C UGAUU

90 80 70 60 50

1. ***novel_MIRNA_390***

10 20 30 40 50 60 70

GUGCU| C U AC A U UUGUUUG U UG GG AUGA

G AC GUCG CUG UUUGGUCGUGAU AUCGCGG UUGC U GG CAUGGGU U

C UG UAGC GAC AAACCAGCACUA UAGCGCC AACG G UU GUACCCA U

UUUUG^ U U GA A C UUCUGGA - GU A- AAAU

. 130 120 110 100 90 80

1. ***novel_MIRNA_553***

10 20 30 40 50

A| A A - UCUAA ACC

CUUCUCCUAGAAUUGCCACUU AU CUUUUUUG CAAUUCUUA UUAG \

GAAGAGGGUCUUAACGGUGAG UA GAAAAAAC GUUAAGGAU AAUC U

C^ G - U C---- AAU

100 90 80 70 60

1. ***novel_MIRNA_639***

10 20 30 40 50

GAAACCAA--| U AAC A UUUU

AUC UCAGG AGUUGAAGAUGAGAUAUUG ACGAAGA \

UAG AGUCC UCAACUUUUACUCUAUAAC UGUUUUU A

ACAACACCCA^ U CUA C CUUU

100 90 80 70 60

1. ***novel_MIRNA_641***

10 20 30 40

UACUU| A C U A C G A

ACUA UCCUUC GUC CAUAAUAUAA AA GUUUUU ACACUA \

UGAU AGGAAG CAG GUAUUAUAUU UU CAAAAA UGUGAU U

AUUUU^ G A U C A A G

90 80 70 60

1. ***novel_MIRNA_652***

10 20 30 40

-| G G A UGUAU

UCCGUUC GAAUUACUUGUC CG AAAUGGAUGUAUCUAGA \

AGGCAAG UUUAAUGAACAG GC UUUACCUACAUAGAUCU U

G^ G A C UGAUU

90 80 70 60 50

1. ***novel_MIRNA_669***

10 20 30 40 50

C| AACUU

CUGACGUACUGCAGGGUGCAGUUGGGUCACUGACAGGUGGGCC G

GACUGCAUGACGUCCCACGUCAACCCGGUGACUGUCCACCCGG C

-^ GCUUU

90 80 70 60

1. ***novel_MIRNA_678***

10 20 30 40 50

AUGUUAUAC - U C - .-AAA| GCU U A

AGAAGGGG AGCC UGG GCA GUGGU GCU GCCU GUGACC U

UCUUUCCC UCGG GCC CGU CAUCG UGA UGGG CACUGG G

GAAAUUAUA G - A A \ ---^ ACU - A

. 190 180 170 70 60

80 90

.-CCUGGAAA --- G

CAGCCU CUUACA A

GUCGGA GGAUGU A

\ -------- AAG A

110 100

120 130 140

CGUACUAUAGA .-AA U ACC

CCC AG GGUCGG C

GGG UC CCAGCC U

A---------- \ -- - CCU

150

G

GC C

CG A

A

160

1. ***novel_MIRNA_692***

10 20 30 40 50

AC AA U U A ---| C AC AU

GCGAGGA UUCC GCGGGG CGA CUGGGAACACAUGG GAUA GA CGCUUG \

CGCUCCU GAGG CGCCCC GCU GACUCUUGUGUACU CUAU CU GCGAGC U

GC CC U C G CAA^ U CA AG

. 110 100 90 80 70 60

1. ***novel_MIRNA_810***

10 20 30 40 50

----| A UC - G U AAU A UCA

UGUC GGGGGUG AGUUUCUU CAGAA AGCAUG CAUUUUCUGG GA GGC A

AUAG CCCCCAC UCAAAGAA GUCUU UUGUGC GUAAAAGACC CU CCG A

CAAC^ - CA A G C CUC A UCA

. 110 100 90 80 70 60

1. ***novel_MIRNA_866***

- A CC A | A

CAUU UAGUGUAGUCUCGU UCUUGCUAAGAUAU UUUAAA--UCCUC G

GUAA AUUAUAUCAGAGCA GGAGCGGUUCUAUA AGGUUU AGGAG C

C C UA C \ ^ A

120 110 100 90 50

60 70

AACAUACAG AAA

CUUA G

GAAU G

AG------- AUA

80

***(18) novel_MIRNA_1009***

10 20 30 40 50

-| C A U C UGUAU

ACUCC UCCGUUCCGA UUACUUGUCGCAGA AUGGAUGUAU UAGA \

UGAGG AGGCAAGGUU AAUGAGCAGCGUCU UACUUAUAUA AUUU U

A^ A G U U UGAUU

100 90 80 70 60

***(19) novel_MIRNA_1154***

10 20 30

-| UC C CA

GUACCC AUAAAAAGAAUGA CUCAUUGUCUU \

CAUGGG UAUUUUUCUUACU GAGUAACAGAA A

C^ GA A UC

70 60 50 40

(20) novel_MIRNA_1255

10 20 30 40 50 60 70 80

UGC A -| GCU UAAUUACCCCCCACU U -------- CGU

CUUUCCACAGCUUUCUUGA CUUC UCUUGC CUCCU CUC CUGUCUCUCCCU UUCUCUC U

GAAGGGUGUCGAAAGAACU GGAG AGAACG GAGGA GAG GACAGGGAGGGG GAGAGAG G

AAA G A^ AC- C-------------- - UAAGUACA AUG

150 140 130 120 110 100 90

***(21) novel_MIRNA_1304***

10 20 30 40 50

UUUU| C C U GAUAA GCU

UUA CACUG CGGUGAGUGCUU AUAUCUUCUU UUUUG A

AAU GUGAC GCUACUCACGAA UAUAGAAGAA AGAAC A

UUAU^ A A C AGA-- ACA

. 90 80 70 60

***(22) novel_MIRNA_1322***

10 20 30

C| U A A UG

UCUUCCUA CUAGAG AGCACAU CAAAAAAAUU G

AGAAGGAU GAUCUC UCGUGUA GUUUUUUUGG C

A^ C C G UU

70 60 50 40

***(23) novel_MIRNA_1372***

10 20 30 40

U| C U U C C GCCGAG AA

UCU CGUGGUU CCGGU CCAC UCGGC GCC UCCCAUG \

AGA GUAUCAA GGCCA GGUG AGCCG CGG GGGGUGC C

U^ C U C - A UA---- GG

90 80 70 60 50

***(24) novel_MIRNA_1401***

10 20 30 40 50 60

AU| AC C U C ACA A A CG

UGUAC UC C CCGU CCAUAAUAUAAGAUGUUUUUUGACACUA UA UGU AGA \

AUAUG AG G GGCA GGUAUUAUAUUCUAUAAAAAACUGUGAU AU ACA UCU U

UU^ A- C - A GUG C G UU

120 110 100 90 80 70

***(25) novel_MIRNA_1419***

10 20 30 40 50 60 70

G U - CC C --- .-CCAACA .-GCAA| C

UUAAAAGUG UA GGAACAACAACCGCAUUUUC CAUGG UCUCAU UAGGAUAGAUCCAA GCC UU U

AAUUUUCAC AU CCUUGUUGUUGGCGUAAAAG GUACC AGGGUA AUCCUAUCUAGGUU CGG AG A

- U C AC A AAA \ ------ \ ----^ G

190 180 170 160 150 140 130 80

90

AA C

GUAGC G

UAUCG A

GA A

100

110

ACCCC UCC

CCAUG A

GGUAC U

CUU-- UAC

120

***(26) novel_MIRNA_1544***

10 20 30 40

A---| UGCG U U AU

GCAAAGAU AAGAGCGCGAAGGAUU GCAGA ACUCCGUAAA \

CGUUUCUG UUCUUGUGCUUCUUAA CGUCU UGAGGUAUUU C

UUGA^ CAA- C C CA

90 80 70 60 50

***(27) novel_MIRNA_1560***

10 20 30 40 50 60 70 80

- U U U C C AUU .-AAUAA UU---| AA C

GAA AUA UGUAAACC UCACAAAUU CCUUGAUUAUUU AUAGCGCU UUAA AUGA UGUAUUGGA AAC \

UUU UAU ACAUUUGG AGUGUUUAA GGAACUAGUAAG UAUCGCGA AGUU UACU AUAUAACCU UUG A

G C U - - U AAU \ ----- UAUAC^ G- A

. 270 260 250 240 230 100 90

110

.-G G

ACUU A

UGAA A

\ - A

120 130 140 150 160

.-AAAAAAAUACA A UG AUUG- AGAAAGA

GUC U UAU UGUUUGACUU A

CGG A GUA ACAAAUUGAA A

\ ----------- A GU GAAAA AAAGUAG

190 180 170

200 210

AG GCUU U

AUGAAU UGC U

UACUUA ACG A

A- AGU- U

220

***(28) novel_MIRNA_1613***

10 20 30 40

UCCUUCCU -| A U

GGUGGGCGUACG GAACACGGGUG UUUUUC U

CUACCCGCAUGC CUUGUGCCCAC AAAAGG A

ACCCUUUC A^ - G

. 70 60 50

***(29) novel_MIRNA_1617***

10 20 30 40 50 60 70 80

C U C -| G UC AUA GG UC U C - CUUG

AUAACC CUUGAAU UUGG GAAAAA CUGCAUAAC CA AAUU AAACC AU UU UAUCCCA AAAUUG C

UAUUGG GAACUUA AACC CUUUUU GACGUAUUG GU UUGA UUUGG UA AA AUGGGGU UUUAAC A

C U U A^ A GA A-- AG UA U A C ACAA

. 150 140 130 120 110 100 90

***(30) novel_MIRNA_1686***

10 20 30 40 50 60

- - A .-UUACAUAUA .-UAUUUUUGAAA| A GA

GAUCUG CUUCUCAUCUGAAGACU GUUUA AGCA GUUUG GA G

CUAGAU GAAGAGUAGAUUUCUGA CAAAU UCGU CGAAC CU A

A G A \ --------- \ -----------^ A AG

170 160 150 70

80

AGA---- GUG

UCCC C

AGGG A

UACUACA AAG

100 90

110 120

ACAACUA AA CCA

CAAU UUCUU \

GUUA GAGAA C

CGCCAG- CC CUU

140 130

***(31) novel_MIRNA_1737***

10 20 30 40 50

AU| CAA A CCAA

GGG CCUUCA CCCUCUUUAGUUCAAUCAAGUGGAUUAAAUUU A

CCC GGAGGU GGGAGGAAUCAAGUUAGUUUACUUGAUUUAGA A

CU^ ACG G CAUU

100 90 80 70 60

***(32) novel_MIRNA_1744***

10 20 30 40 50 60 70 80 90 100 110

CG U CUC G AUC GGAA C UG CCAC UU-----| A

GCUAC CU CGUCC GGUGUAUAAGUCAUUCGCGUAGUUUUAGGUC GAUUUGA UUAAAUAUGUGUUAUAUGU A AAAAGUAUA UAGA UCC C

UGAUG GA GCAGG CCACAUAUUCAGUAAGCGCAUCAAGAUCCAG UUAAAUU GAUUUAUACAUAAUAUACA U UUUUUAUAU AUCU AGG A

CA - A-- G CUA GAUA C GU AUAA UUGAUGU^ C

220 210 200 190 180 170 160 150 140 130 120

***(33) novel_MIRNA_1875***

10 20 30 40 50

G U - U A C A CC-| GA-- AG

CUC C GG AGC GC GUUGAG UGAGAUUACCCCAUAC UG GCGUC \

GAG G CC UCG CG CAACUC ACUCUAAUGGGGUAUG AC CGUAG C

- U U C C A C CGC^ AUGC CU

100 90 80 70 60

***(34) novel_MIRNA_2079***

10 20 30 40 50

C --- CU U G A .-GU -|G

GC GCC GGAG CUCUGGUUGGUCUGU UUUGUUUCAAAC AGGUG CAGG C U

CG CGG CCUC GAGGCCAACCAGACA AAGCAGAGUUUG UCCAC GUCC G A

A AUA U- U A C \ -- U^C

. 120 110 100 90 60

70

U- CUCUC

GC \

CG U

GU UUGUU

80

***(35) novel_MIRNA_2133***

10 20 30 40

G UA -| UA

AAUUACUUGUCUUGGAUUUGUCUAGAUA GAUGUAUCUAG ACU C

UUAAUGAACAGAAUCUAAACAGAUCUAU CUACAUAGAUC UGA U

- GC G^ UU

90 80 70 60 50

***(36) novel_MIRNA_2185***

10

A .-A| CU

CUGCAUU GUGAU G

GAUGUGA CAUUA G

- \ -^ UU

180 20

30 40 50 60 70 80 90

U A G A .-GAGAA AUU- GUGA

CAAG GAAAGCAAUGUCAGA GAGUUUUAGCA CAGUCUGAA GCUG AUGUAUAU \

GUUC CUUUCGUUACGGUUU CUCGAAGUUGU GUCAGACUU CGAC UAUAUGUG G

A C A C \ ----- GGCU AGAG

170 160 150 140 110 100

120

A GA

GUAUAUUC \

UAUGUGAG G

A AG

130

***(37) novel_MIRNA_2191***

10 20 30 40 50 60 70 80 90

- G A CG- G GA-------------- UGC- -| AAUUCAUGC A AA CA AAAGU

CA CCUAC UUGAUCCCA UCUAAG CCUGGAGCAGCGUAGACGU GGGGU GUUGAAAU AUUG UCU GCC UC UUCA \

GU GGAUG AGCUGGGGU AGAUUC GGAUCUCGUCGUAUUUGCA CCCCA UAACUUUA UAAC AGA CGG AG AAGU C

C G C ACA A AAAAAAACACAACCCC CCAU U^ --------- - -- AG CAUAC

200 190 180 170 160 150 140 130 120 110

***(38) novel_MIRNA_2567***

10 20 30 40 50

G| AU A C U

CGUUUC UUCAUCAAUUUCUGUCUAACUAUUGUGCA UGUACC UG G

GUAAAG AAGUAGUUAAAGACAGAUUGAUAACACGU ACAUGG AC A

A^ -- G A C

90 80 70 60

***(39) novel_MIRNA_2745***

10 20 30 40 50 60 70 80 90 100 110

UCUC G C GU G C A ------- -- .-AACGACAACAUA| UGAA AA

CAUAU AA UG CUUGCUUAAUUUU UAGUCUUCUGGAUAUAUGCUUA AUAUAAUAA UAAU GUGA AGGUAGAUCA--UAG AUCUUUAGUCU CA \

GUAUA UU AC GAACGAAUUAGAG AUUAGAAGACUUGUGUACGAAU UAUAUUAUU AUUA UACU UCCAUCUAGU AUC UAGAGAUCAGA GU U

AAAU G C UG G C G GGAUAGC GU \ \ ------------^ UG-- AU

350 340 330 320 310 300 290 280 270 130 120

140 150

AGAACA - AACCAGAC

CUAUA CG \

GGUAU GC A

***(40) novel_MIRNA_2917***

10 20 30 40 50

G U -| G U AAU A UCA

GGGGUG CAGUUUC UUCAGAA AGCAUG CAUUUUCUGG GA GGC A

CCCCAC GUCAAAG AAGUCUU UUGUGC GUAAAAGACC CU CCG A

- C A^ G C CUC A UCA

100 90 80 70 60

***(41) novel_MIRNA_3314***

10 20 30 40 50 60 70

GU AA A AGCGUU-- -| A G CAU AG

UGCUACCAUGAAGAGCGCGGGCAGCACAACCG GGC CU GCGUA GG GG GCUG CGC \

GCGAUGGUACUUCUCGCGCCCGUCGUGUUGGC CUG GA CGCGU CC CC CGGC GCG C

CU -- C CCAUACUU G^ A G U-- UA

140 130 120 110 100 90 80

***(42) novel_MIRNA_3417***

10 20 30

A GAACUU - -| C GAGU

UUGAUU UCCAA CGGA CUCAUUC AU \

AACUAA GGGUU GCUU GGGUAAG UA A

G AGCAAC C A^ U ACAA

70 60 50 40

***(43) novel_MIRNA_3607***

10 20 30

A GAACUU - -| C GAGU

UUGAUU UCCAA CGGA CUCAUUC AU \

AACUAA GGGUU GCUU GGGUAAG UA A

- AGCAAC C A^ U GCAA

70 60 50 40

***(44) novel_MIRNA_3791***

10 20 30 40

AA - AA---| AAAAAG A UGGA

GA UGAAA ACCAAA CUCUGCCCUUCC UCUCU \

CU ACUUU UGGUUU GAGACGGGAGGG AGAGA U

AA G CCCUG^ CCG--- - UAGA

90 80 70 60 50

***(45) novel_MIRNA_4191***

- A C C UU .-A| AAUC A AUCUA

CUGCU UGCA ACGAUACU UUGGACGAUUC GGACGAUA UGCA AG CC \

GAUGA GUGU UGCUAUGA GACCUGCUAAG UCUGCUAU ACGU UC GG U

U A C C UC \ -^ GGA- A AAAUA

. 130 120 110 100 70

80

A-------- AG

CAC \

GUG C

ACAAAUUAG AC

90

***(46) novel_MIRNA_4740***

10 20 30

U| GCAAUUAAAC UUU

UGAUU UCGGCUCAAUCUUUUUU \

ACUAG AGCCGAGUUAGAAAAAA U

-^ AUAAAAAUA- UCU

70 60 50 40

***(47) novel_MIRNA_5134***

10 20 30 40 50 60 70 80 90

G - A UGGAGCAU - - GA- C U ACA -------- .-CA| U

CGAGGA CCCGACAGAGUUGGGC GUAUCCA GGG GCG GAGG AGGGCAU CCCAUA GA AAGUAGU CGAGC GGGU G

GUUUCU GGGUUGUCUCAACCCG CAUAGGU CCC CGC CUCC UCCCGUA GGGUAU CU UUCAUCG GCUCG CCCG C

- G C -------- A G ACC - C CGC UCCCGCUG \ --^ U

220 210 200 190 180 170 160 150

100

.-A - AU

GUGC CGCG \

CACG GUGU C

\ - A CA

120 110

130

GA C C

CCA CG C

GGU GC A

AG U C

140

***(48) novel_MIRNA_5169***

10 20 30 40 50 60 70 80 90

- G U C AU--------- UG GCA .-AAUAACAUA| CAA AAG

CUUAAUUUU UAAUCUUCUGGA AUAUGCUUG AUAUAAUAA GAUG AAGGUAGA UAGAAC AUCUUUAGUCU GC G

GAAUUAGAA AUUAGAAGAUUU UGUAUGAAU UAUAUUAUU CUAC UUCCAUCU GUUUUG UAGAGAUCAGA UG U

C G U C GAUUAGAAGAU UA A-- \ ---------^ --- AGG

. 300 290 280 270 260 250 240 110 100

120 130

.-AGAA .-AAGA CA

UGC UGUGU \

ACG ACACA C

\ ---- \ ---- UA

160

140

A------ AC

CA A

GU A

AUGAAAG AC

150

170 180 190 200

AAAAA U CC G G CA

UGGUAAA GGAGCU UGACUU UAGAUUAU U \

AUCAUUU CUUCGA ACUGAG AUCUAGUA G A

ACC-- U -- - G GA

230 220 210

***(49) novel_MIRNA_5172***

10 20 30

G --- - ---| C

AUCCCUUUU UUGGCGUCCCCAUGUC CC CC U

UAGGGAAAG AACUGUAGGGGUACGG GG GG G

- GAA C UGU^ U

70 60 50 40

***(50) novel_MIRNA_5542***

10 20 30 40 50 60 70

CGU AA A AGCGUU-- -| A G CAU AG

UGCUACCAUGAAGAGCGCGGGCAGCACAACCG GGC CU GCGUA GG GG GCUG CGC \

GCGAUGGUACUUCUCGCGCCCGUCGUGUUGGC CUG GA CGCGU CC CC CGGC GCG C

UCU -- C CCAUACUU G^ A G U-- UA

140 130 120 110 100 90 80

***(51) novel_MIRNA_5552***

10 20 30 40

GU GAUAUUAUAC G UG UUC .-A| G

GAUU CUCCC GACCAGAACUUCU U AG GGG G

CUAA GGGGG CUGGUUUUGGAGG G UC CCU A

G- AGACGCA--- A GU UAA \ -^ A

120 110 100 90 50

60 70

AUCAA AUCAA

GCUUG C

CGAAC C

CUAA- AAUUA

80

***(52) novel_MIRNA_5598***

10 20 30

AUUCCUCUUU| A AA

ACCG AGAAGCCUGUGCUCGA \

UGGU UUUUCGGGCACGAGCU U

UAUGCAAAAC^ C AA

60 50 40

***(53) novel_MIRNA_5756***

10 20 30

-| AAAA -- A UGGA

CA AAAG CUCUGCCCUUCC UCUCU \

GU UUUC GAGACGGGAGGG AGAGA U

U^ GG-- CG - UAGA

60 50 40

***(54) novel_MIRNA_5816***

10 20 30 40

GU- G A ----------|UC CG

CUCUGGUUGGUCUGU UUUGUUUCAAAC AGGUG G AGGCGUA \

GAGGCCAACCAGACA AAGCAGAGUUUG UCCAC C UCCGUGU U

UCU A C GUCGUUGUUU^UC CC

100 90 80 70 60 50

***(55) novel_MIRNA_5831***

10 20 30 40 50 60 70

U CU G U A G- A U AAAUUU .-A| AG

GCUCC UGCGA CUCGGCCGUUGGAUC UUGAU CAGCGGACAU UG GG GCUCC UUUGCA GA C

CGAGG ACGCU GAGUCGGCAACCUAG AGCUA GUCGCCUGUA GC CC CGGGG GGACGU CU C

- C- G - G GA - U ACCU-- \ -^ CC

160 150 140 130 120 110 80

90

AAGAA CU

GUCA \

CAGU A

G---- AA

100
