# Supplementary material for: Identification and Temporal Expression Analysis of Conserved and Novel MicroRNAs in the Leaves of Winter Wheat Grown in the Field
Source: Front Genet. 2019 Sep 4;10:779. doi: 10.3389/fgene.2019.00779 (PMC6737308; doi:10.3389/fgene.2019.00779)
Supplement: Supplementary file 10 [file Table_10.docx]

**Supplemental file 2. Foldback structure of wheat putative MIRNAs predicted by mfold server**

(1) ***novel_MIRNA_1153***

10 20 30 40

-| U AUAA

UCCGUUCGGAAUUACUUGUC CGGAAAUGGAUGUAUCUAGA A

AGGCAAGCCUUAAUGAACAG GUCUUUACCUACAUAGAUCU A

G^ C GCAU

90 80 70 60 50

**(2) *novel_MIRNA_1202***

10 20 30

-| C C U

CCUGAA GUCUUCUUCAAGUA UCCACUUUUUUUUUC U

GGACUU UAGAAGAAGUUCAU AGGUGGAAAAAGAGG G

U^ U A C

70 60 50 40

**(3) *novel_MIRNA_1250***

10 20 30 40

-| UC UC CUAC

ACUC UCUAUCCCAUAAUAUAAGAGCGUUUU ACA \

UGAG AGGUAGGGUAUUAUAUUCUCGCAAAA UGU A

A^ GA GA AAUU

80 70 60 50

***(4) novel_MIRNA_1306***

10 20 30

AAACCAAAAAA| -- A - G

AAG CUCUGCCCUUCC UCUCU UC A

UUC GAGACGGGAGGG AGAGA AG U

AAAAACAAG--^ CG - U A

70 60 50 40

***(5) novel_MIRNA_1327***

10 20 30 40

C U -------| UC UUU

UC GGUUGAAUUUGUCCAUAGCAUCAGCCA AUCC UGU U

AG CCAACUUAAAUAGGUAUCGUGGUCGGU UGGG GCA U

U C AGCAUCA^ UA UGU

90 80 70 60 50

***(6) novel_MIRNA_1356***

10 20 30

-| C C UC

CCUGAA GUCUUCUUCAAGUA UCCACUUUUUUUUC \

GGACUU UAGAAGAAGUUCAU AGGUGGAAAAAGAG G

U^ U A GC

70 60 50 40

***(7) novel_MIRNA_1481***

10 20 30 40 50

A| G GAAA CU C AUGA

GUGUGAAACACUGU UGAGAGAAGUCAUAAGUGU CA CU CG U

CACACUUUGUGACA AUUCUCUUCAGUAUUCACA GU GA GC G

-^ G AAC- C- A AGUA

100 90 80 70 60

***(8) novel_MIRNA_1559***

10 20 30 40

AAG| U G

UACUCCCUCCGUCCCAUAAUAUAAGAGCGUUUUU ACACUA \

AUGAGGGAGGCAGGGUAUUAUAUUCUCGCAAAAA UGUGAU U

AUG^ C G

90 80 70 60 50

***(9) novel_MIRNA_1576***

10 20 30 40 50

AU .-CAA C G A | A GCAU

CACCGG GUUCCAGA GAUGCAG CCUUC UCAAG--GCGG GG G

GUGGUC CAAGGUCU CUACGUC GGAAG AGUUC UGCC CC G

UU \ --- A A G \ ^ - AAGA

. 140 130 120 60

70

.-AGUGA U

CGACC A

GUUGG G

\ ----- C

80

90 100

AUU AAAG GG

UGGC GUGGA A

ACUG CGCCU A

U-- GUUA AA

***(10) novel_MIRNA_1615***

10 20 30 40 50 60 70 80 90 100

UUU C A U GC AU CUC AAC .-CAA| A A- ACGAC

GAA CAAAUAUGACUAGGUAUUGUACUC UAUAAAAAG UUG CA GAAUGAUU GUU UUCGGGG AUAAG CA AUUUAUG A

CUU GUUUAUACUGGUUCAUAACGUGAG AUGUUUUUC AAU GU CUUACUGA UAA AGGCCCC UGUUC GU UAAGUAC A

UGU A C U UU CC AAA CAU \ ---^ - GA GAUAU

220 210 200 190 180 170 160 150 120 110

130

A--------- CA

UCUAG U

GGGUC U

AAUUUCUAAA UU

140

***(11) novel_MIRNA_1620***

10 20 30 40

UUU AU-- U- A GA--------- GUC UA- | U

UGCU UCAGCU GUUGGUA GCC GC UCCA AG A

GCGA GGUCGA CGAUUAU CGG CG AGGU UC A

GCU GAUU CU C AAAGACCAUUG AAA UAA ^ A

. 90 80 70 60 50

***(12) novel_MIRNA_1634***

10 20 30 40 50 60 70 80 90 100 110 120 130 140 150

-| C C CAGACUG UA CAU A A G UG A - CAA C ACUACA

AACGCCUCACAGAUAG CGUUACACGCCUGGC AGCGUUGCACCC CC AAAAUUGCUAAGU UGUGCAG GCCU AGAGCUA GCGC CACUGUAUAGUGUGGC CCUAGCU CU GCG UGCACUAGUGGUUGC \

UUGCGGAGUGUCUAUC GCGAUGUGCGGACUG UCGCAGCGUGGG GG UUUUAAUGAUUCA ACACGUC CGGA UCUCGAU CGCG GUGAUAUGUCACAUCG GGAUCGA GA CGC ACGUGAUCACCAACG A

G^ C C CACACUA C- AUC G C A GU C A UC- A AAGUAA

300 290 280 270 260 250 240 230 220 210 200 190 180 170 160

***(13) novel_MIRNA_1786***

10 20 30 40 50 60 70 80 90 100

- C A --| A UGUGAU - A C A U CA A

GCAA GCCUCACAGAUAG CGCUACACGCCUGGCCAGCGU CAU CCCG CC AAAAAUU CUAAGUCAGUGUGCAG GCC GA AGCUAGGCGC CACU U

UGUU CGGAGUGUCUAUC GCGAUGUGCGGACCGGUCGCA GUA GGGU GG UUUUUAA GGUUCAGUUACACGUU CGG UU UCGAUCCGCG GUGA A

A A C AC^ - UGGGAC A C U A C AC C

210 200 190 180 170 160 150 140 130 120 110

***(14) novel_MIRNA_1791***

10 20 30 40 50 60

G G -| UU CACU GU- CCUA

ACGAUACU UUGGACGAUUCAGAGACGAUA UGU AAUC GGCUGU UGCA G

UGCUAUGA AACCUGCUAAGUCUCUGCUAU ACG UUAG CUGGUA AUGU U

G G C^ U- U--- GAU UUCC

. 110 100 90 80 70

***(15) novel_MIRNA_1818***

10 20 30 40 50 60 70 80 90

U GAA- G-- A U .-AUAAGAAGAU - CG--- .-GA .-A - ----| AAG U AA

GGAAAG CAUGGCCAG GAA GGAGG GGA GAAG AACA AGGG AAUGUC UUGUU GGGUCUU AC G CAAGA \

CCUUUU GUAUUGGUC CUU CCUUC CUU UUUC UUGU UUCC UUGCAG AACAA CCCAGAA UG C GUUCU A

U AACA GAA C - \ ---------- U AAAAA \ -- \ - U GAAC^ GAA - GG

280 270 260 220 210 190 120 110 100

130 140

CGU AU A

GGCUUUA--GCAG UC A

CCGGGAU CGUC GG U

U-- \ GU C

180 150

160

A-------- CA

GUUU A

CAAG C

CUAUUAUCU AA

170

AU

CGGCA \

GUCGU C

CU

200

230

UG--- AU

CAGC AGUU \

GUCG UCAG G

UUUAA CG

250 240

***(16) novel_MIRNA_1826***

10 20 30 40 50 60 70 80 90 100

C C C C A UUG A CAGUC A .-GAGGAGUUCAGA .-A| ---- G

UG CAG CAAGACGAGGGUGAUCAUAAACU CUGGGU AUCAUG GAC CC CAGG ACAUGCC GCGG GAGGUUCA AG G

AC GUC GUUUUGCUCUCACUAGUAUUUGA GACCCG UAGUAU UUG GG GUCC UGUACGG CGCC UUUCAGGU UC A

U U C C G --- A ACGAA A \ ------------ \ -^ ACGU G

220 210 200 190 180 170 160 110

120 130

CAUG U U

GUGUAU C U

CACAUG G C

G--- - G

140

150

AGA A

CC \

GG A

UA- A

***(17) novel_MIRNA_1890***

10 20 30 40 50

-| C U GA AAACUAA

UCCGUCU AAAAUACUUGUCAUCAAAA GGAUGAAAAG AUGUAUCUA \

AGGCAGG UUUUAUGAACAGUAGUUUU CUUAUUUUUC UACAUAGAU A

G^ C U AC CUACAUA

110 100 90 80 70 60

***(18) novel_MIRNA_2129***

10 20 30 40 50 60 70

AG CC- G C - .-CUAAAC GCCC UG- .-AAAAAAAAAA A-| CC

GGCG UAAUCAGC GC CCACCCA ACAGCA GAA AUA UUUAUC GGA GC A

UUGC AUUGGUUG UG GGUGGGU UGUUGU CUU UAU GAAUGG CCU UG U

GA ACU A U C \ ------ AACC UUA \ ---------- AA^ UA

190 180 170 130 120 80

90 100

A U CAU

GU GAAGGC G

CA CUUUUG A

A U CGC

110

140

--------- - AA

CUGA AAU U

GACU UUG A

ACAAAUAAA G AU

160 150

***(19) novel_MIRNA_354***

10 20 30 40

G| C U AC UAACA

UGCCGUAACUGCACCUGCAGUUGGG CA UG AUGUGGGCC G

ACGGCAUUGACGUGGACGUCAACCC GU AC UACACCCGG A

-^ A U CA UCGGC

90 80 70 60 50

***(20) novel_MIRNA_450***

10 20 30 40 50 60 70

GCAU U C A AC C G CC - C U-- -| GA

AAAG UUGACCA GU UAUGGAGAAAA AUUUA AUCUA AA AC AAA AUAUAUCAU AGAUU CGU C

UUUU AACUGGU CA AUAUCUCUUUU UAAAU UAGAU UU UG UUU UAUGUAGUA UUUGA GUA A

AACC U U A AA A G A- A C UAC U^ GA

150 140 130 120 110 100 90 80

***(21) novel_MIRNA_465***

10 20 30 40 50

- C UA U-| GUA

CUCCCUCUGUUCCGAAUUACUUGUCG AGG UGGAUGUAUCUA AU U

GAGGGAGGCAAGGUUUAAUGAGCAGC UCU ACCUACAUAGAU UG U

U U UG CU^ AUU

100 90 80 70 60

***(22) novel_MIRNA_523***

10 20 30 40 50 60

G| UCUU A U - U CCC AUCCA U UUC

GGAGC CAC CCGUGGAUGUCAUCG GGCCGUACA UGAA CGUCC AC GC CUG A

UCUCG GUG GGCACCUACAGUAGU CCGGCAUGU ACUU GCAGG UG CG GAC A

-^ ---- C - U U --- AAA-- - UUU

120 110 100 90 80 70

***(23) novel_MIRNA_546***

10 20 30 40 50

GCU AA U C A - A U--| GC

CGUU UU GCUUCAAGCCUUU GGAAU GU GUA ACUGCAC GC \

GCAA AA CGAAGUUCGGAAG CCUUA CG CAU UGACGUG CG A

AGU CG U U - C C CCU^ AU

100 90 80 70 60

***(24) novel_MIRNA_658***

10 20 30 40 50

A| A C A UAACA

GUGCC UAACUGCACCUGCAGUUGGG CAGUG CAUGUGGGCC G

CACGG AUUGACGUGGACGUCAACCC GUUAC GUACACCCGG A

-^ C A C UCGGU

90 80 70 60

***(25) novel_MIRNA_84***

10 20 30 40

G -----------| C G

AGGUACUCCCUCCGUCCCAAAAUAAGUGUCU CAA UUUGUACUA C

UUCAUGAGGGAGGCAGGGUUUUAUUCACAGA GUU AAAUAUGAU U

G ACUCGAAUCAU^ U C

100 90 80 70 60 50

***(26) novel_MIRNA_929***

10 20 30 40 50

C| A UA UG

CAACGAGACCGACUGC GCGGGAGCAAAAAUUAUGCCAUUGAAUUCG C \

GUUGUUCUGGUUGAUG CGCCUUUGUUUUUGAUAUGGUGACUUAAGU G A

U^ C GA AA

100 90 80 70 60

***(27) novel_MIRNA_947***

10 20 30 40 50 60

- U C AGAA--| AAAAUAAAUA

UUCGGUGAGAAACGA GUUUUUUGUGGCUUG GUAAAAAAGAUAA AUGC \

AAGCCACUUUUUGCU CAAAAAACACCGAAC CAUUUUUUCUGUU UACG G

A C A AAAGAC^ AAGUAAUGUU

. 120 110 100 90 80 70
