# Supplementary figures and images for: Identification and Temporal Expression Analysis of Conserved and Novel MicroRNAs in the Leaves of Winter Wheat Grown in the Field
Source: Front Genet. 2019 Sep 4;10:779. doi: 10.3389/fgene.2019.00779 (PMC6737308; doi:10.3389/fgene.2019.00779)

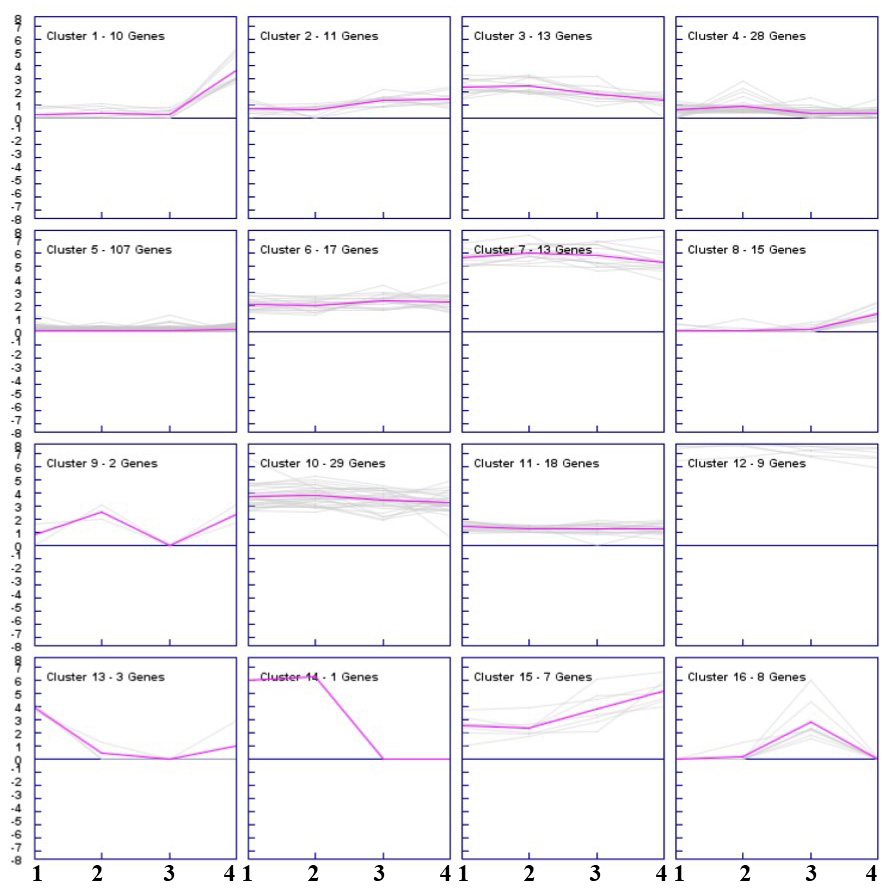

Supplement: Supplementary file 11 [file Image_1.jpeg]
